# Abscisic acid and sucrose regulate tomato and strawberry fruit ripening through the abscisic acid‐stress‐ripening transcription factor

**DOI:** 10.1111/pbi.12563

**Published:** 2016-05-04

**Authors:** Haifeng Jia, Songtao Jiu, Cheng Zhang, Chen Wang, Pervaiz Tariq, Zhongjie Liu, Baoju Wang, Liwen Cui, Jinggui Fang

**Affiliations:** ^1^ Key Laboratory of Genetics and Fruit DevelopmentHorticultural CollegeNanjing Agricultural UniversityNanjingChina

**Keywords:** tomato fruit, strawberry fruit, transcription factor ASR, sucrose, abscisic acid

## Abstract

Although great progress has been made towards understanding the role of abscisic acid (ABA) and sucrose in fruit ripening, the mechanisms underlying the ABA and sucrose signalling pathways remain elusive. In this study, transcription factor ABA‐stress‐ripening (ASR), which is involved in the transduction of ABA and sucrose signalling pathways, was isolated and analysed in the nonclimacteric fruit, strawberry and the climacteric fruit, tomato. We have identified four ASR isoforms in tomato and one in strawberry. All ASR sequences contained the ABA stress‐ and ripening‐induced proteins and water‐deficit stress‐induced proteins (ABA/WDS) domain and all ASR transcripts showed increased expression during fruit development. The expression of the *ASR* gene was influenced not only by sucrose and ABA, but also by jasmonic acid (JA) and indole‐3‐acetic acid (IAA), and these four factors were correlated with each other during fruit development. ASR bound the hexose transporter (*HT*) promoter, which contained a sugar box that activated downstream gene expression. Overexpression of the *ASR* gene promoted fruit softening and ripening, whereas RNA interference delayed fruit ripening, as well as affected fruit physiological changes. Change in *ASR* gene expression influenced the expression of several ripening‐related genes such as *CHS*,*CHI*,* F3H*,*DFR*,*ANS*,*UFGT*,*PG*,*PL*,*EXP1/2*,*XET16*,* Cel1/2* and *PME*. Taken together, this study may provide new evidence on the important role of ASR in cross‐signalling between ABA and sucrose to regulate tomato and strawberry fruit ripening. The findings of this study also provide new insights into the regulatory mechanism underlying fruit development.

## Introduction

Mechanisms underlying fruit development and ripening are an important research area. In the past decade, studies on fruit development were mainly focused on the physiological and biochemical metabolism of fruit development (Kourmpetli and Drea, 2014; Kumar *et al*., 2014; Obrucheva, 2014). Based on the patterns of respiration and ethylene production during fruit maturation and ripening, the fruits were classified into two categories: climacteric and nonclimacteric (Alexander and Grierson, 2002; Barry and Giovannoni, 2007; Klee and Giovannoni, 2011; Ren *et al*., 2010; Rodrigo *et al*., 2006). In recent years, comprehensive studies on climacteric fruits such as tomato and banana (Adams‐Phillips *et al*., 2004; Alba *et al*., 2005; Beekwilder *et al*., 2004; Fei *et al*., 2004; Gupta *et al*., 2006) have been conducted. However, the mechanism underlying the ripening of nonclimacteric fruit differed from that of climacteric fruit, and ethylene showed no effect on the ripening of nonclimacteric fruit (Chervin *et al*., 2004; Trainotti *et al*., 2005). Therefore, the nonclimacteric fruit‐ripening mechanism has not been very well elucidated. In our previous studies, we determined that the plant hormone, abscisic acid (ABA), played an important role in strawberry fruit ripening by influencing strawberry fruit softening, aroma, anthocyanin biosynthesis and growth (Given *et al*., 1988; Jia *et al*., 2011). However, others factors also contributed to strawberry fruit ripening. Methyl jasmonate also played a role in fruit cell wall metabolism and anthocyanin metabolism (Mukkun and Singh, 2009; Rudell *et al*., 2002). There are multiple signalling pathways associated with fruit ripening, and in addition, we have shown that sucrose also acts as a signal involved in strawberry fruit ripening (Jia *et al*., 2013). Collectively, these results suggest that additional work needs to be conducted to establish a network of molecular mechanism associated with nonclimacteric fruit ripening. Unlike strawberry, tomato is a climacteric fruit where fruit softening and ripening is controlled by the ethylene signal transduction pathway (Griesser *et al*., 2008; Iusem *et al*., 1993; Johnson and Ecer, 1998; Kevany *et al*., 2007, 2008). However, the role of other factors, such as ABA or sucrose, remains elusive.

Previous reports have showed that sucrose and ABA regulate the expression of some genes together (Jossier *et al*., 2009; Rolland *et al*., 2006; Tang *et al*., 2009). Sucrose controls the expression level of the *ApL3* gene, which encoded, ADP glucose focal phosphorylase (AGPase) in tomato leaf and fruit, whereas ABA does not. However, when both sucrose and ABA were present at the same time, they significantly induced the expression of the *ApL3* gene (Li *et al*., 2002). Sucrose and ABA also induced the activity of sucrose synthase (*SS*) in rice (Tang *et al*., 2009). Exogenous sucrose promoted the maturity of strawberry fruit, which was achieved by regulating ABA levels (Jia *et al*., 2013). Therefore, sucrose and ABA together regulate the expression of several genes that confer multiple signal transduction crosstalk activity. (Finkelstein and Gibson, 2001; Jossier *et al*., 2009; Rolland *et al*., 2006). To identify the precise function of ABA and sucrose during strawberry fruit ripening, the *Fragaria *×* ananassa* ABA‐stress‐ripening (*FaASR*) gene in strawberry, which could be induced by both ABA and sucrose, was characterized (Chen *et al*., 2011).

ABA‐stress‐ripening (ASR) proteins are small and basic proteins that could be induced by ABA, stress and ripening. These are strongly hydrophilic due to its high levels of His, Glu and Lys (Cakir *et al*., 2003; Henry *et al*., 2011; Konrad and Bar‐Zvi, 2008; Wang *et al*., 2005; Yang *et al*., 2005). These proteins consist of two main highly conserved regions: a short N‐terminal consensus containing a typical stretch of six His residues in an eight‐amino acid sequence that might constitute a Zn‐binding site and a longer C‐terminal region made of at least 70 amino acids. Analysis of ASR proteins in Loblolly pine and melon has shown that a putative nuclear targeting signal is present at the C‐terminus of ASR protein (Canel *et al*., 1995; Carrari *et al*., 2004; Chang *et al*., 1996). The expression of the *ASR* gene varies among different species, organs and conditions (Golan *et al*., 2014). Although the ASR gene has been well characterized, the mechanism of ASR in response to fruit ripening at the molecular level is not well understood. There have been various reports of ASRs involved in plant senescence and fruit development, as well as in response to water deficit, salt, cold and limited light (Frankel *et al*., 2006; Kalifa *et al*., 2004; Shkolnik and Bar‐Zvi, 2008). However, information on the role of the *ASR* gene and protein in tomato and strawberry fruit development and ripening is limited (Frankel *et al*., 2006, 2007; Jeanneau *et al*., 2002; Kalifa *et al*., 2004; Konrad and Bar‐Zvi, 2008; Liu *et al*., 2010; Shen *et al*., 2005; Shkolnik and Bar‐Zvi, 2008). Therefore, the lack of knowledge associated with the function of ASR and its molecular regulatory mechanisms during fruit ripening necessitates further investigation.

To analyse the role of transcription factor ASR in fruit ripening in strawberry and tomato, followed by the synthesis of a fruit ripening regulatory model, we focused on various parameters, which included physiology and ASR gene expression patterns in response to ABA, sucrose, jasmonic acid (JA) and auxin during tomato and strawberry fruit development. In this study, a tobacco transient expression system, gene silencing and gene overexpression techniques in strawberry and tomato fruit were used to verify the biological function of transcription factor ASR.

## Results

### Morphological and physiological changes during strawberry and tomato fruit development

Based on the changes in fruit size and colour, the fruit development process of tomato cultivar ‘*cv*. Ailsa Craig’ was divided into eight stages: SIG, MIG, BIG, MG, B, T, MR and OR at about 20, 25, 29, 33, 37, 42, 45 and 48 days after anthesis, respectively (Figure S1a). Strawberry fruit development was divided into seven stages: SG, LG, DG, WT, IR, PR and FR for 7, 13, 16, 19, 22, 25 and 28 days after anthesis (Figure S1b), respectively. In this study, we observed distinct morphological and physiological changes between climacteric (tomato) and nonclimacteric (strawberry) fruit during the ripening process. It was observed that the tomato fruit developed rapidly under regular cultivation conditions (about 50 days from anthesis to ripening), and the period from strawberry anthesis to ripening was about 30 days. In terms of fruit size, the tomato fruit had a steady phase from fruit stages MG (33 days after anthesis) to T (42 days after anthesis) (Figure S1a), whereas strawberry fruit size continuously increased without a steady phase (Figure S1b). In tomato, ABA content increased from the SIG (20 days) to the T and showed the highest level in the T (42 days) stage, but then gradually decreased in the OR (48 days) stage (Figure S2a), and the *SlNCED1* gene played an important role in ABA synthesis in the tomato fruit (Figure S3a). Ethylene content showed a peak occurring after the ABA peak (Figure S2a). In strawberry, ABA content continuously increased from the SG (7 days) to the FR (28 days) stage (Figure S2e). The expression patterns of the *FaNCED1* and *FaNCED3* genes were similar to the changes in ABA contents during strawberry fruit development (Figure S3b). Ethylene content rapidly increased before the WT (19 days) stage and then slowly increased, and after the WT (19 days) stage, markedly increased again (Figure S2e). However, ABA promoted fruit‐ripening process, whereas ethylene had no significant effect on this particular progress (Figure S4), indicating that, unlike tomato, ABA, but not ethylene, played an important role in strawberry ripening.

In the strawberry fruit, the sucrose content showed a more rapid rate of increase than the other two glucose and fructose (Figure S2f). On the other hand, in the tomato fruit, glucose and fructose contents continuously increased during fruit development, whereas sucrose content did not significantly change (Figure S2b). The expression level of sucrose was correlated to the sucrose transporter (*FaSUT*) and sucrose phosphate synthase (*FaSPS*) genes in strawberry, and the sucrose degradation‐related acid invertase gene (*FaAI*) and double functional gene, sucrose synthase gene (*FaSS*), declined with strawberry fruit development, thereby allowing the accumulation of high sucrose (Figure S5a). However, in tomato, the expression levels of the *SlSPS* gene and the *SlSUT* gene decreased with tomato fruit development and that of the *SlSS* gene showed same variation. Only the *SlAI* gene showed an increase in the expression during fruit development (Figure S5b). These findings suggested that sucrose is easily degraded and led to a low sucrose content during tomato fruit development. The anthocyanin contents of the tomato and strawberry fruits showed a similar trend (Figure S3c; g). Fruit firmness dramatically changed from the B (37 days) to the MR (45 days) (Figure S3d) stages in tomato fruit, whereas that of strawberry varied from the DG (16 days) to the WT (19 days) stages (Figure S3 h). In tomato, total soluble solid content significantly changed from the BIG (29 days) to the MG (33 days) stages (Figure S3d), whereas in strawberry, the changes in total soluble solid content were correlated to the fruit development process (Figure S3 h).

### Cloning and computational analysis of tomato and strawberry *ASR* genes

**Figure 1:**
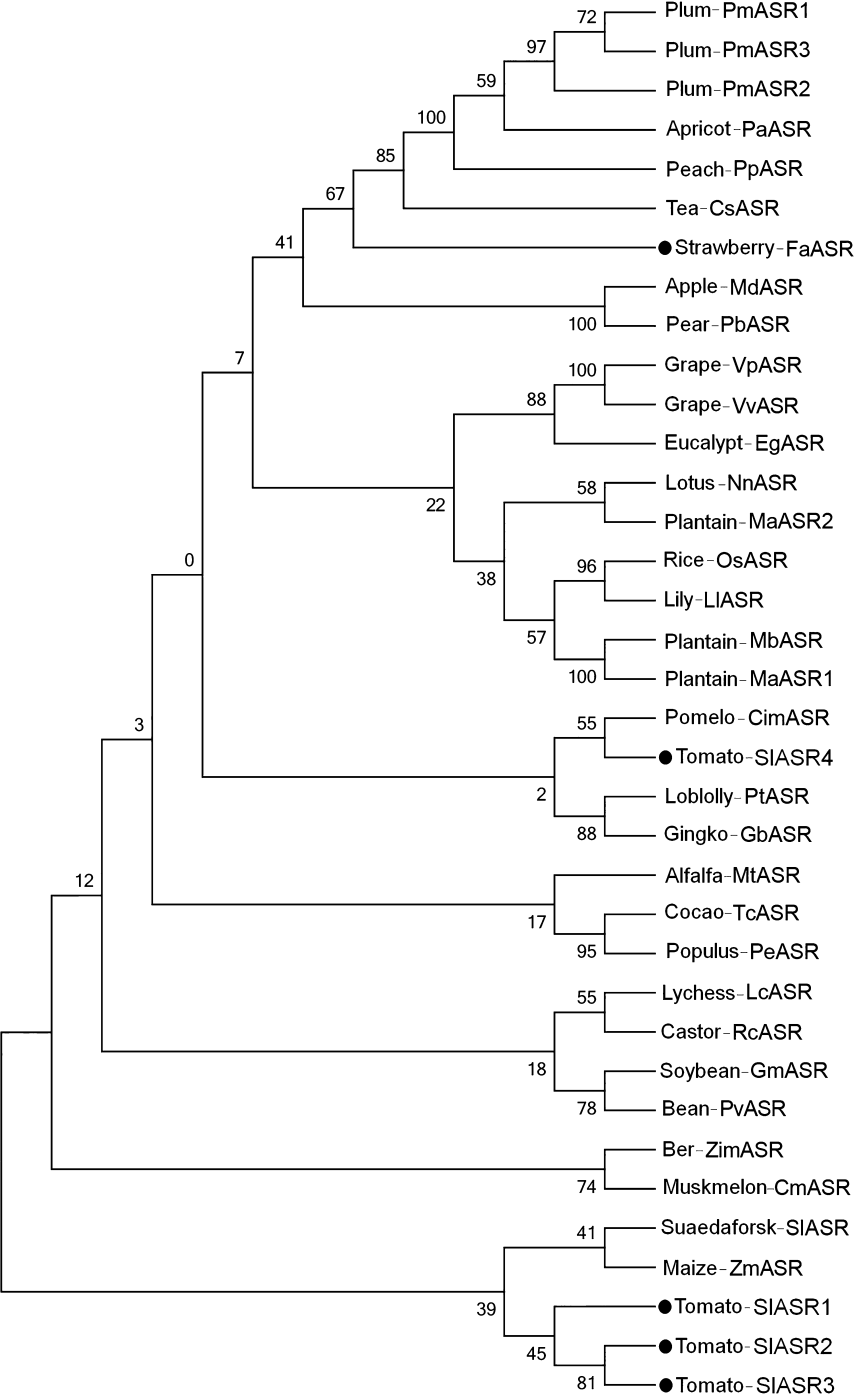
Phylogenetic tree of some *ASR* gene. Studied genes are indicated by a black circle. All sequence data may be found in GenBank; accession numbers are shown in Table S2.

To clone the *ASR* genes of tomato (*SlASR*) and strawberry (*FaASR*), the grape ASR protein sequence (GenBank Accession No. AF281656) was used to conduct a BLAST search in the strawberry gene library (https://strawberry.plantandfood.co.nz/index.html) and the tomato gene library (http://solgenomics.net/). Four tomato proteins showing a high level of identity with gene locil SL2.31sc04135, SL2.31sc04135, SL2.31sc04135 and SL2.31sc04135 were identified, and one strawberry protein encoded by gene locus08120 was detected. Specific primers (Table S1) were designed to amplify the encoding sequences of tomato and strawberry fruits by RT‐PCR and then sequenced. The CDS of four candidate *ASR* genes products in tomato were 333, 345, 327 and 894 bp in size and that in strawberry was 579 bp in length. They all included an open reading frame that encoded a deduced protein of 110, 114, 108 and 297 amino acids in tomato and 192 amino acids in strawberry (Figure S6). Phylogenetic analysis also showed highly similar sequence homology to several other ASR proteins from different plant species (Figure 1; Table S2; 3).

Two highly conserved regions were identified: a small N‐terminal consensus of 18–20 amino acids that contained a typical stretch of six His residues within an 8‐amino acid sequence and a large C‐terminal region of at least 80 amino acids (Figure S6). Checking for specific sequences in SlASR and FaASR using the BLOCS method (http://bloCs.fhcrc.org) revealed the presence of one ABA/WDS signature, which was previously described in ABA stress and ripening‐induced proteins (Mbeguie *et al*., 1997) and in water‐deficit stress‐induced proteins (Padmanabhan *et al*., 1997). In addition, the 3′ end of the C‐terminal part of ASR contained a putative signal for nuclear targeting (Shen *et al*., 2005; Figure S6).

### *ASR* gene expression in tomato and strawberry

**Figure 2:**
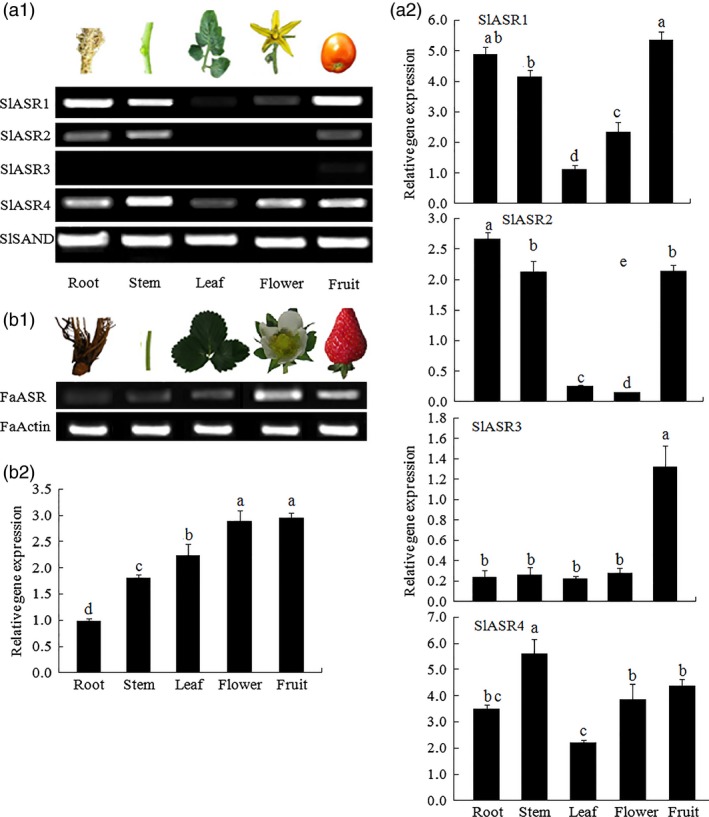
*ASR* gene expression in different tissues of tomato and strawberry. (a1) The *SlASR1*,* SlASR2*,* SlASR3* and *SlASR4* genes expression level were determined by semiquantitative (Sq) RT‐PCR (26 cycles) and qRT‐PCR in the root, stem, leaf, flower and ripening fruit of tomato, and the reference gene was *SlSAND*. (b1) The *FaASR* gene expression level was determined in the root, stem, leaf, flower and ripening fruit of strawberry by SqRT‐PCR (26 cycles) and qRT‐PCR, and the reference gene was *FaActin*. Vertical bars represented standard deviations (SD) of means (*n* = 3). Different letters indicated a statistical difference at *P *<* *0.05 as determined by Duncan's multiple range test.

**Figure 3:**
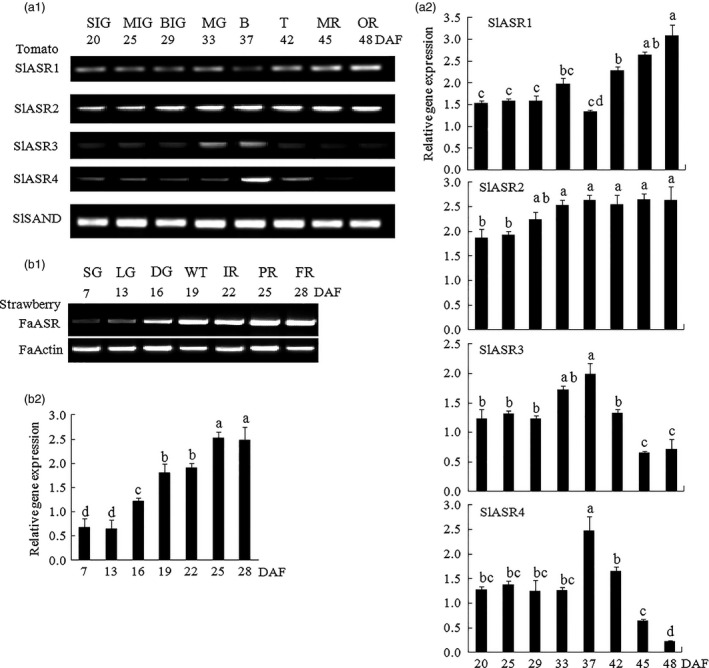
Semiquantitative (Sq) RT‐PCR and qRT‐PCR analyses of the mRNA level in the receptacles of fruits during developmental stages. (a1) Four members of *ASR* genes *SlASR1*,* SlASR2*,* SlASR3* and *SlASR4* expression levels were determined by SqRT‐PCR (26 cycles) (a2) and qRT‐PCR in the process of tomato fruit development, and the reference gene was *SlSAND*. SIG (20 days): small immature green; MIG (25 days): middle immature green; BIG (29 days): big immature green; MG (33 days): mature green; B (37 days): breaking; T (42 days): turning; MR (45 days): mature red; OR (48 days): over red. (b1) *FaASR* gene expression level was determined by SqRT‐PCR (26 cycles) (b2) and qRT‐PCR in the process of strawberry fruit development, and the reference gene was *FaActin*. SG (7 days): small green; LG (13 days): large green; DG (16 days): degreening; WT (19 days): white; IR (22 days): initial red; PR (25 days): partial red; FR (28 days): full red. Vertical bars represented standard deviations (SD) of means (*n* = 3). Different letters indicated a statistical difference at *P *<* *0.05 as determined by Duncan's multiple range test.

*ASR* gene expression pattern was measured in tomato and strawberry, and the results showed that the four *SlASRs* genes in tomato had different expression levels. The tissue expression level of the *SlASR1* and *SlASR4* genes were higher than those of *SlASR2* and *SlASR3* (Figure 2a1,a2); the expression level of the *SlASR1* gene was higher in the fruit, and *SlASR4* was higher in the stems. Higher levels of expression of the *SlASR2* gene were observed in root, stem, and fruit, whereas lower levels of expressions were detected in the leaf and flower. Higher level of expression of the *SlASR3* was observed in the fruit, whereas lower levels were detected in other tissues (Figure 2a1,a2). The *FaASR* gene was expressed in all the strawberry tissues studied with higher levels in the flower and fruit (Figure 2b1,b2). To investigate whether the *SlASRs* and *FaASR* genes were involved in fruit ripening, its mRNA expression levels were determined by semiquantitative PCR and quantitative RT‐PCR (qRT‐PCR) using the tomato eight‐stage fruits and strawberry seven‐stage fruits, respectively. The results showed that in the tomato fruit, the mRNA expression levels of the *SlASR1* gene did not vary from 20 to 37 day, but from the 42 day, the expression levels rapidly increased until to fruit ripening (Figure 3a1,a2). On the other hand, the level of the expression of the *SlASR2* did not vary from 20 to 48 day after flowering. For the *SlASR3* and *SlASR4* genes, its expression levels increased from 20 to 37 day, which was then followed by a decrease (Figure 3a1,a2). In the strawberry fruit, the *FaASR* mRNA expression levels were extremely low during the SG (7 days) and LG (13 days) fruit stages and then rapidly increased during fruit development and finally remained at an extremely high level at the FR (28 days) stage (Figure 3b1,b2). Taken together, the expression of the *SlASR1* gene was more relevant with fruit development and higher expression level in fruit compared to the *SlASR2*,* SlASR*3 and *SlASR*4 genes. Shkolnik and Bar‐Zvi (2008) also presented that tomato SlASR1 could abrogate the response to ABA and glucose in *Arabidopsis* by competing with ABI4 for DNA binding; therefore, the *SlASR1* gene was used in the subsequent analyses.

**Figure 4:**
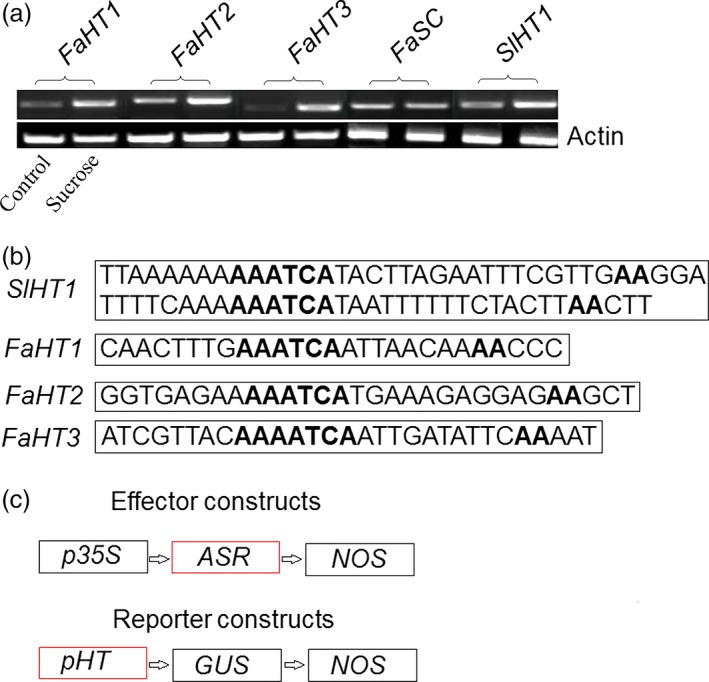
The promoter of hexose transporter (*pHT*). (a) Sucrose (100 mm) was used to induce *HTs* genes expression level in strawberry and tomato. There are three *HTs* genes in strawberry: *FaHT1*,* FaHT2* and *FaHT3*, and one of *SlHT1* in tomato. In strawberry, one of sugar carrier (SC) gene was also treated by sucrose (100 mm). These were repeated three times and similar results were obtained. (b) The promoter of *HTs* from tomato (one *pSlHT*) and strawberry (three *pHTs*:* pFaHT1*,* pFaHT2* and *pFaHT3*) were isolated and analysed by software of PLACE (http://www.dna.affrc.go.jp/PLACE/signalscan.html). The bold font represented the sucrose box, and the primers of the promoters could be found in Table S1. (c) The method of vector construction. The promoter 35s of pBI121 were replaced by the promoter of *HTs* genes from strawberry and tomato to form pBI121‐*pSlHT1*/*pFaHT1*‐GUS, respectively, or the GUS of pBI121 was replaced by the *SlASR1* or *FaASR* gene to form pBI121‐35s‐*SlASR1*/*FaASR*.

**Figure 5:**
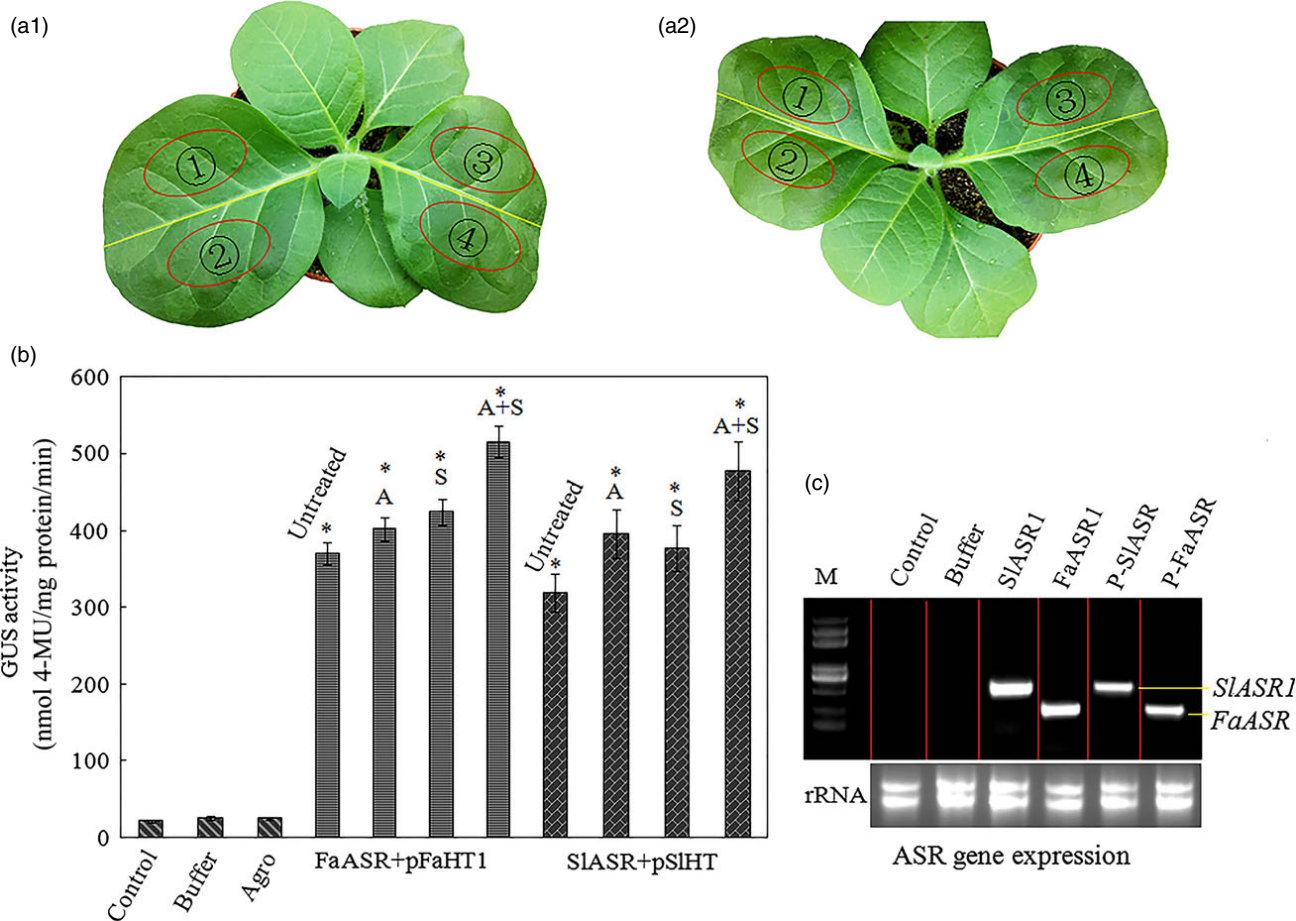
Test of interaction of transcription of *SlASR1* or *FaASR* with the promoter of hexose transporter gene *HT* from tomato or strawberry using tobacco transient expression system. (a) The tobacco leaf were 2 weeks old; *Agrobacterium* was introduced by dispensable 1‐mL syringe infiltration in young leaves of tobacco plants and transformed in a stable manner with the mixture: (a1) 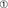: not anything infiltration; 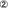: only infiltration buffer; 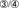: mixture of *FaASR* + *pFaHT1*‐GUS. (a2) 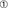: *Agrobacterium* containing pBI121‐35s‐*FaASR/SlASR1* plasmid only; 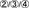: mixture of *SlASR1 *+* pSlHT*‐GUS. Every treatment was repeated for ten plants. Tobacco leaves of infiltration were collected 2 days later, and they were used to determine (b) GUS activity: total protein was extracted from the leaves of noninfiltration (control), buffer, *Agrobacterium* (*FaASR* or *SlASR1*), and *FaASR* + *pFaHT1* or *SlASR* + *pSlHT* infection (untreated). Sucrose (S, 100 mm), abscisic acid (ABA) (A, 50 μm) and sucrose (S, 100 mm) + ABA (A, 50 μm) were used to treat *FaASR* + *pFaHT1* or *SlASR1 *+* pSlHT*‐GUS infection leaves; 8 h later, the leaves were collected for determination of GUS activity. (C) *ASR* gene expression level in the infiltration leaves. Total RNA was isolated from the leaves of control, buffer, *Agrobacterium* (*FaASR* or *SlASR1*) and mixture of *FaASR* + *pFaHT1* (p‐*FaASR*) or *SlASR1 *+* pSlHT*‐GUS (p‐*SlASR*)‐infiltrated leaves; *Agrobacterium* (*FaASR* or *SlASR1*) was used as a control to ensure the exogenous *ASR* gene expression successfully in tobacco leaves. These were repeated four times. Asterisks indicated statistically significant differences at *P* < 0.05 as determined by Student's *t*‐test.

As a transcription factor, ASR could bind a *cis*‐acting element to regulate downstream gene expression (Kalifa *et al*., 2004). Because ASR could bind to sucrose box in the promoter of the hexose transporter gene (*HT*) in grape (Cakir *et al*., 2003), the promoter of the tomato and strawberry *HT* gene was selected to further verify the biological function of ASR. We tested the interaction between the ASR protein and the promoter of the tomato and strawberry *HT* gene, using a transactivation assay. We found one hexose transporter gene, *SlHT1*, in tomato, and three FaHT genes (*FaHT1*,* FaHT2* and *FaHT3*) in strawberry, and all were sucrose inducible (Figure 4a). We also detected a sugar carrier gene, *FaSC*, in strawberry that was not sucrose inducible (Figure 4a). Isolation and analysis of its promoter region of the *SlHT1* and *FaHT* genes showed that these contained a *cis*‐acting element in the sucrose box, whereas the promoter of the sugar carrier gene in strawberry did not contain the sucrose box, which was suggestive of its role in sucrose‐inducible gene expression (Figure 4b). And then the mixture of pBI121‐35s‐ASR and pBI121‐*pSlHT1*/*pFaHT1*‐GUS (Saumonneau *et al*., 2012; Figure 5a) were injected into the tobacco leaves, after 2 days, the expression levels of the *ASR* genes were assessed by semiquantitative(Sq) RT‐PCR. GUS activity was also detected, and the results showed that in tobacco leaves infiltrated with mixture of pBI121‐35s‐ASR and pBI121‐*pSlHT1*/*pFaHT1*‐GUS, GUS activities were six‐ to sevenfold higher than that of the untreated leaves. On the other hand, in tobacco leaves infiltrated with pBI121‐35s‐ASR alone or with infiltration buffer, a slight increase in GUS activity was observed. To confirm the accuracy of our results, ABA (100 μm) and sucrose (100 mm) were also used in the treatment of the tobacco leaves that were infiltrated with a mixture pBI121‐35s‐ASR and pBI121‐*pSlHT1*/*pFaHT1*‐GUS. A slight increase in GUS activity was observed in the treated leaves relative to that in the untreated leaves (Figure 5b). The ASR genes of tomato or strawberry were expressed in the pBI121‐35s‐ASR‐infiltrated leaves (Figure 5c), indicating that transient expressed system was effective. These suggested that the biological function of the transcription factor, ASR, was to induce downstream gene expression, which was enhanced by ABA and sucrose.

### Factors influencing *ASR* gene expression

**Figure 6:**
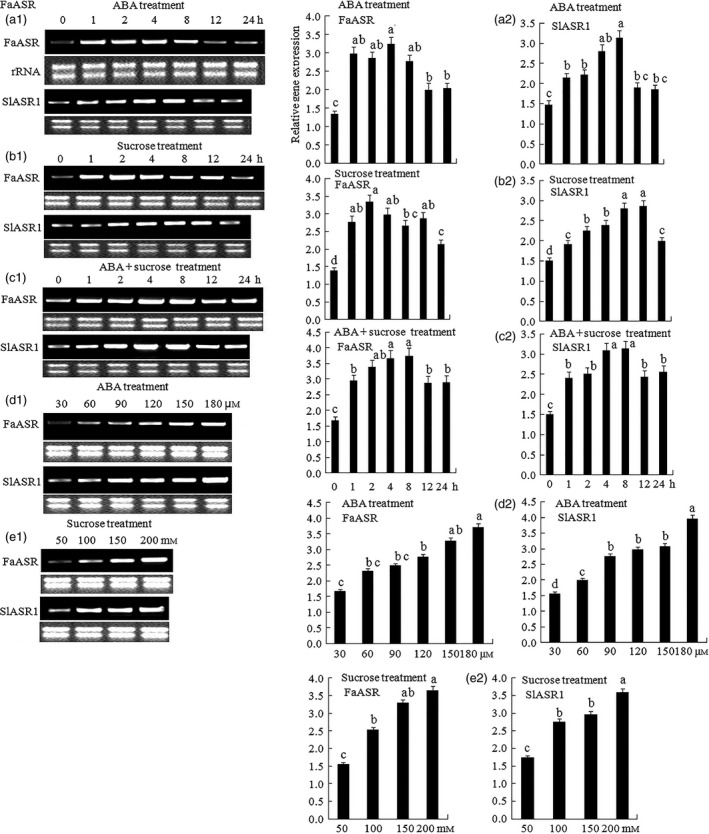
Effect of abscisic acid (ABA) and sucrose on the *SlASR1* and *FaASR* gene expression. Fruits attached to plants were used to inject various reagent. The ABA (60 μm) induced the *SlASR1* and *FaASR* genes expression in different time (0, 1, 2, 4, 8, 12 and 24 h) in tomato and strawberry fruit. (a1) Semiquantitative (Sq) RT‐PCR and (a2) qRT‐PCR analyses were performed to determine the *SlASR1* and *FaASR* genes expression levels. Similarly, sucrose (100 mm) induced the *SlASR1* and *FaASR* genes expression in different time (0, 1, 2, 4, 8, 12 and 24 h). (b1) SqRT‐PCR and (b2) qRT‐PCR analyses were performed to determine the *SlASR1* and *FaASR* genes expression levels. ABA (60 μm)  + sucrose (100 mm) induced the *SlASR1* and *FaASR* genes expression in different time (0, 1, 2, 4, 8, 12 and 24 h). (c1) Sq RT‐PCR and (c2) qRT‐PCR analyses were also performed to determine the *SlASR1* and *FaASR* genes expression levels. Sucrose (4 h) induced the *SlASR1* and *FaASR* genes expression in different concentration (50, 100, 150 and 200 mm). (d1) Sq RT‐PCR and (d2) qRT‐PCR analyses were also performed to determine the *SlASR1* and *FaASR* genes expression levels. ABA (4 h) also induced the *SlASR1* and *FaASR* genes expression in different concentration (30, 60, 90,120, 150 and 180 μm). (e1) Sq RT‐PCR and (e2) qRT‐PCR analyses were also performed to determine the *SlASR1* and *FaASR* genes expression levels. About 1 μg of total RNA was reversed for every sample in the same condition, and they were repeated for two times and got the similar results. The *SlSAND* was used as a reference gene for tomato, and *FaActin* was used as a reference gene for strawberry. Vertical bars represented standard deviations (SD) of means (*n* = 3). Different letters indicated a statistical difference at *P *<* *0.05 as determined by Duncan's multiple range test.

The pattern of *ASR* gene expression indicated that it might be involved in fruit ripening, and thus, its response to ripening‐related factors such as sucrose and ABA during fruit development was investigated. First, strawberry fruits at three developmental stages, namely SG (7 days), LG (13 days) and FR (28 days), were treated with ABA and sucrose to determine which stages rapidly responded to ABA and sucrose. The results showed that fruits at the LG (13 days) and FR (28 days) stages had a stronger response to ABA and sucrose compared to that observed in SG (7d) stage (Figure S7a). For tomato, the SIG (20 days)‐, MG (33 days)‐ and OR (48 days)‐stage fruits were treated with ABA and sucrose, and the results showed that, different to *SlASR2* (Figure S7c), *SlASR3* (Figure S7d) and *SlASR4* (Figure S7e), *SlASR1* expression was induced by ABA and sucrose in the three stages, except for ABA at the OR (48 days) stage (Figure S7b). To further verify the effect of ABA and sucrose on *ASR* gene expression, different concentrations of ABA and sucrose and time treatments were used to assess *ASR* gene expression levels. The results showed that in tomato, in 8 h, the longer the induction time of ABA (Figure 6a1,a2), sucrose (Figure 6b1,b2) and ABA + sucrose (Figure 6c1,c2), the higher the *SlASR1* gene expression levels. After 8 h, the degree of response to ABA and sucrose gradually decreased, but *SlASR1* gene expression levels remained higher than that of the control and was similar to that of strawberry *FaASR* gene expression after treatment with ABA (Figure 6a1,a2), sucrose (Figure 6b1,b2) and ABA + sucrose (Figure 6c1,c2). In the same way, in tomato and strawberry, the higher the induction concentration of ABA (Figure 6d1,d2) and sucrose (Figure 6e1,e2), the higher the *SlASR1* gene expression levels. These results suggested that ABA and sucrose indeed increased the level of *ASR* gene expression.

**Figure 7:**
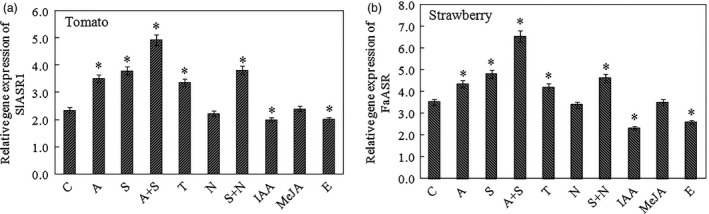
Different treatment on the *SlASR1* and *FaASR* gene expression levels. (a) The abscisic acid (ABA), sucrose, ABA + sucrose, ABA inhibitor nordihydroguaiaretic acid (NDGA), sucrose analogue turanose, methyl jasmonic acid (MeJA), auxin (IAA) and ethephon were used to treat the MG (33 days) fruit of tomato for 4 h, and total RNA was extracted to analyse the *SlASR1* gene expression, and the *SlSAND* gene was used as an internal control. (b) The same treatment was used to analyse the *FaASR* gene expression level in the LG (13 days) fruit of strawberry, and the *FaActin* was used as an internal control. Vertical bars represented standard deviations (SD) of means (*n* = 3). Asterisks indicate statistically significant differences at *P* < 0.05 as determined by Student's *t*‐test. C: control; A: ABA (50 μm); S: sucrose (100 mm); T: turanose (50 mm); N: NDGA (100 μm); IAA (50 μm); MeJA (50 μm); E: ethephon (100 μm).

Second, to identify other factors involved in *ASR* gene expression, sucrose (100 mm) and its analogue, turanose (100 mm), ABA (50 μm) and its inhibitor, NDGA (100 μm), IAA (50 μm), methyl jasmonic acid (MeJA, 50 μm) and ethephon (100 μm) were used to treat strawberry LG (13 days)‐stage (Figure 7a) and tomato MG (33 days)‐stage (Figure 7b) fruits (Dominguez and Carrari, 2015). These results showed that in tomato and strawberry fruits, both sucrose and ABA induced the *ASR* gene expression levels, and sucrose + ABA remarkably promoted the *ASR* expression. Turanose also induced *ASR* gene expression. NDGA had no effect on the levels of *ASR* expression (Figure 7a,b), whereas sucrose + NDGA induced *ASR* expression, which suggests that ABA accumulation might have been blocked by NDGA, and sucrose was activated to induce *ASR* gene expression. These results indicated that sucrose‐modulated *ASR* expression could be mediated via bot an ABA‐dependent and ABA‐independent pathway, and sucrose imparted a stronger effect on *ASR* gene expression compared to ABA during fruit development and ripening (Figure 7a,b). JA is involved in the expression of genes related to fruit cell wall and anthocyanin metabolism, such as *CHS*,* CHI*,* PAL*,* F3H*,* ANS*,* PG1* and *XTH1* (Figure S8), but it had no obvious effect on *ASR* gene expression in tomato and strawberry (Figure 7a,b). IAA inhibited the expression of the ABA biosynthesis gene, *NCED*, and ABA receptor gene, *PYR*, but promoted the expression of the ABA degradation gene, *CYP707A*, which led to a reduction in ABA content. IAA also inhibited the expression of sucrose accumulation‐related genes, *SUT1* and *SPS*, which in turn resulted in a reduction in sucrose content. Taken together, IAA restrained the accumulation of ABA and sucrose, which in turn led to a reduction in the level of *ASR* gene expression (Figure S9c). The other factor that promoted climacteric fruit ripening of ethylene had a negative effect on *ASR* gene expression (Figure 7a,b), indicating that ethylene is also involved in the downstream signal transduction of ABA and sucrose.

Third, to determine the relationship of these ripening‐related factors such as sucrose, ABA, JA and IAA, strawberry fruit was used as a model to analyse the level of expression of metabolism‐associated genes. ABA induced the expression of the JA synthesis gene 12‐oxo‐phytodienoic acid (*FaOPDA1*) and the sucrose transporter gene, *SUT1*, and inhibited the IAA accumulation gene, auxin transporter gene (*FaPIN*), auxin synthesis pathway gene and flavin monooxygenase gene (*FaYUCCA*) (Figure S9a), but had no effect on JA synthesis genes, allene oxide synthase (*FaAOS*) and the sucrose accumulation gene, SPS. Sucrose induced the expression of the ABA synthesis gene, 9‐cis‐epoxycarotenoid dioxygenase (*FaNCED1*), the β‐glucosidase gene, *FaBG3*, ABA degradation ABA 8′‐hydroxylases gene, *CYP707A*, and JA synthesis genes, *FaAOS* and *FaOPDA1*, but had no effect on the IAA accumulation genes, *FaPIN* and *FaYUCCA*, and the ABA receptor pyrabatin resistance gene, *FaPYR* (Figure S9b). IAA down‐regulated the ABA synthesis genes, *FaNCED1*, and the ABA receptor, *FaPYR*, but up‐regulated the ABA degradation gene, *CYP707A,* and another ABA accumulation gene, *FaBG3*. IAA down‐regulated the sucrose accumulation genes, *FaSUT1* and *FaSPS*, but had little effect on JA‐related genes (Figure S9c). JA down‐regulated the IAA accumulation gene, *FaPIN*, and upregulated the *FaBG3* gene for ABA accumulation, but had no effect on ABA, IAA and sucrose accumulation‐related genes (Figure S9d). All these data indicated that sucrose, ABA, JA and IAA had a close relationship during the regulation fruit‐ripening process.

### Silencing of the *ASR* gene inhibits tomato and strawberry fruit ripening

**Figure 8:**
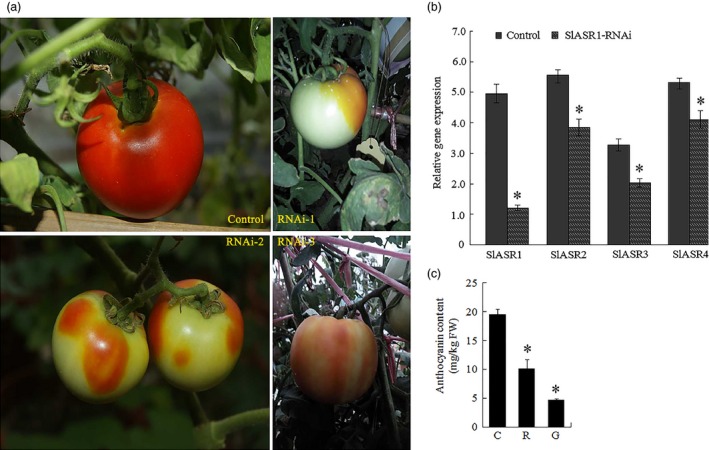
VIGS for the *SlASR1* gene in tomato fruit (RNAi). (a) 33‐day‐old fruits (MG) after flowering still attached to plant were used for inoculation. The control fruit phenotype inoculated with *Agrobacterium* containing the TRV only (control). The RNAi fruit phenotype inoculated with *Agrobacterium* containing TRV carrying a full length of *SlASR1* (RNAi‐1,2,3). (b) *SlASR1*,* SlASR2*,* SlASR3* and *SlASR4* transcriptional level was determined by qRT‐PCR in the control and RNAi fruits. *SlSAND* gene was used as the internal control. Vertical bars represented standard deviations (SD) of means (*n* = 3). (c) The total anthocyanin content was measured in the control and RNAi fruits. C: control fruit; R: red part of *SlASR1‐* RNAi fruit; G: green part of *SlASR1‐* RNAi fruit; Asterisks indicated statistically significant differences at *P* < 0.05 as determined by Student's *t*‐test.

**Figure 9:**
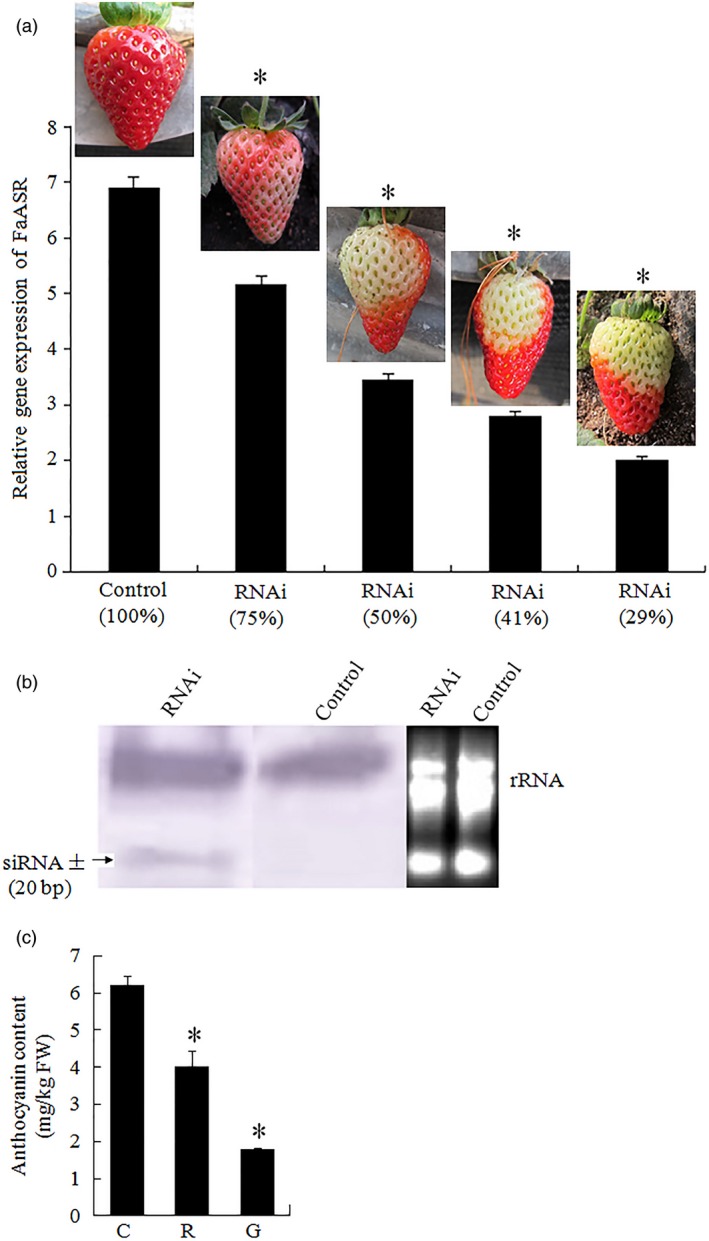
Silencing of *FaASR* by VIGS in strawberry fruit. (a) Two‐week‐old fruits attached to the plant were infiltrated with *Agrobacterium* containing TRV alone (control), or TRV carrying a fragment of *FaASR* (RNAi). Photographs of the infiltrated fruit were taken 2 weeks after infiltration. qRT‐PCR analysis of the transcriptional level of *FaASR* in the receptacle of both control and various RNAi fruits was conducted. *FaActin* gene was used as an internal control. Vertical bars represented standard deviations (SD) of means (*n* = 3). (b) Detection of siRNA (approximately 20 bp) specific to the *FaASR* gene in the control and RNAi fruits. rRNA was the loading control for the RNA samples stained with ethidium bromide. (c) The total anthocyanin content was measured in the control and RNAi fruits. C: control fruit; R: red part of *FaASR‐* RNAi fruit; G: green part of *FaASR‐* RNAi fruit; Asterisks indicated statistically significant differences at *P* < 0.05 as determined by Student's *t*‐test.

Thirty fruits attached to ten independent greenhouse‐grown plants were injected with the *SlASR1‐*RNAi (*SlASR1‐*RNA interfere) vector. After 15 days, the surface of control fruit turned full red (Figure 8a), whereas the surface of the 90% of *SlASR1‐*RNAi fruits did not completely ripen (Figure 8a). To validate the inhibition of the *SlASR1* gene at molecular level, real‐time PCR analysis was performed. The results showed that the *SlASR1* transcripts were markedly down‐regulated in the *SlASR1*‐RNAi fruit compared to that in the control fruit (Figure 8b), together with the other three genes, *SlASR2*,* SlASR3* and *SlASR4* (Figure 8b). On the other hand, anthocyanin content was also down‐regulated in the *SlASR1‐*RNAi fruit (Figure 8c). Similar to tomato, 35 fruits attached to ten independent plants were injected with the *FaASR‐*RNAi (*FaASR‐*RNA interfere) vector and molecular analysis was performed by using real‐time PCR and siRNA techniques. The phenotype of the 90% of *FaASR*‐RNAi fruits involved the absence of colour compared to the control, and the strawberry fruit showed different phenotypes due to the strength of the *FaASR* gene expression silencing (Figure 9a). Molecular analysis indicated that the 500‐bp TRV‐RNA1 (Figure S11a) and 300‐bp TRV‐RNA2 (Figure S11b) were present in the *Agrobacterium*‐mediated TRV‐inoculated fruit, but not in the fruit inoculated with *Agrobacterium* alone. *FaASR*‐related siRNA for specific RNA silencing was also detected in the *FaASR*‐RNAi fruit, but not in the control fruit (Figure 9b), and the total anthocyanin content *FaASR*‐RNAi was lower than that in the control (Figure 9c). Taken together, the *ASR* gene was successfully silenced in the strawberry and tomato fruits, and anthocyanin content was down‐regulated, thereby leading to a delay in the development of the red colour in the fruits.

### Overexpression of the *ASR* gene promotes tomato and strawberry fruit ripening

**Figure 10:**
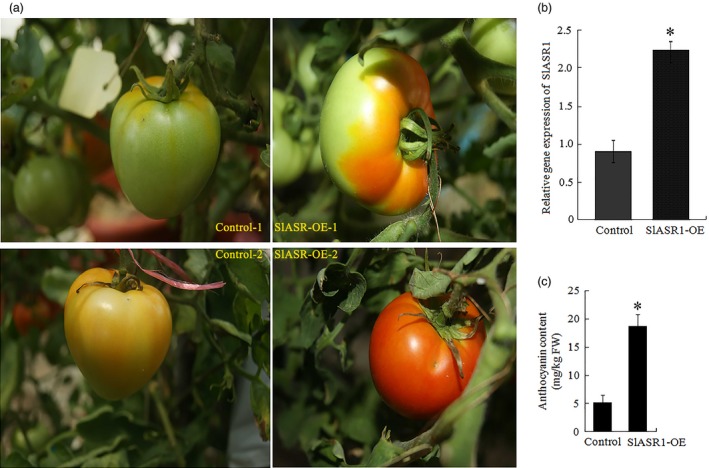
*SlASR1* overexpression (OE) accelerated tomato fruit ripening. (a) The influence of *SlASR1‐* OE on the time course of fruit ripening. *SlASR1* expression was performed as described in the materials and methods section. The *SlASR1*‐OE construct was generated by cloning the coding region of *SlASR1* into the pBI121 vector and placing it under the control of the cauliflower mosaic virus (CaMV) 35S promoter. The *SlASR1* construct was injected into fruits in the BIG (29 days) fruit, and empty pBI121 was used as a control. Six (control‐1, or *SlASR1*‐1) and 12 (control‐2, or *SlASR1*‐2) days after, injection was indicated. (b) qRT‐PCR analysis of *SlASR1* expression was performed in the *SlASR1*‐OE and control fruits. *SlSAND* gene was used as the internal control. Vertical bars represented standard deviations (SD) of means (*n* = 3). (c) The total anthocyanin content was measured in the control and *SlASR1*‐OE fruits. Asterisks indicated statistically significant differences at *P* < 0.05 as determined by Student's *t*‐test.

**Figure 11:**
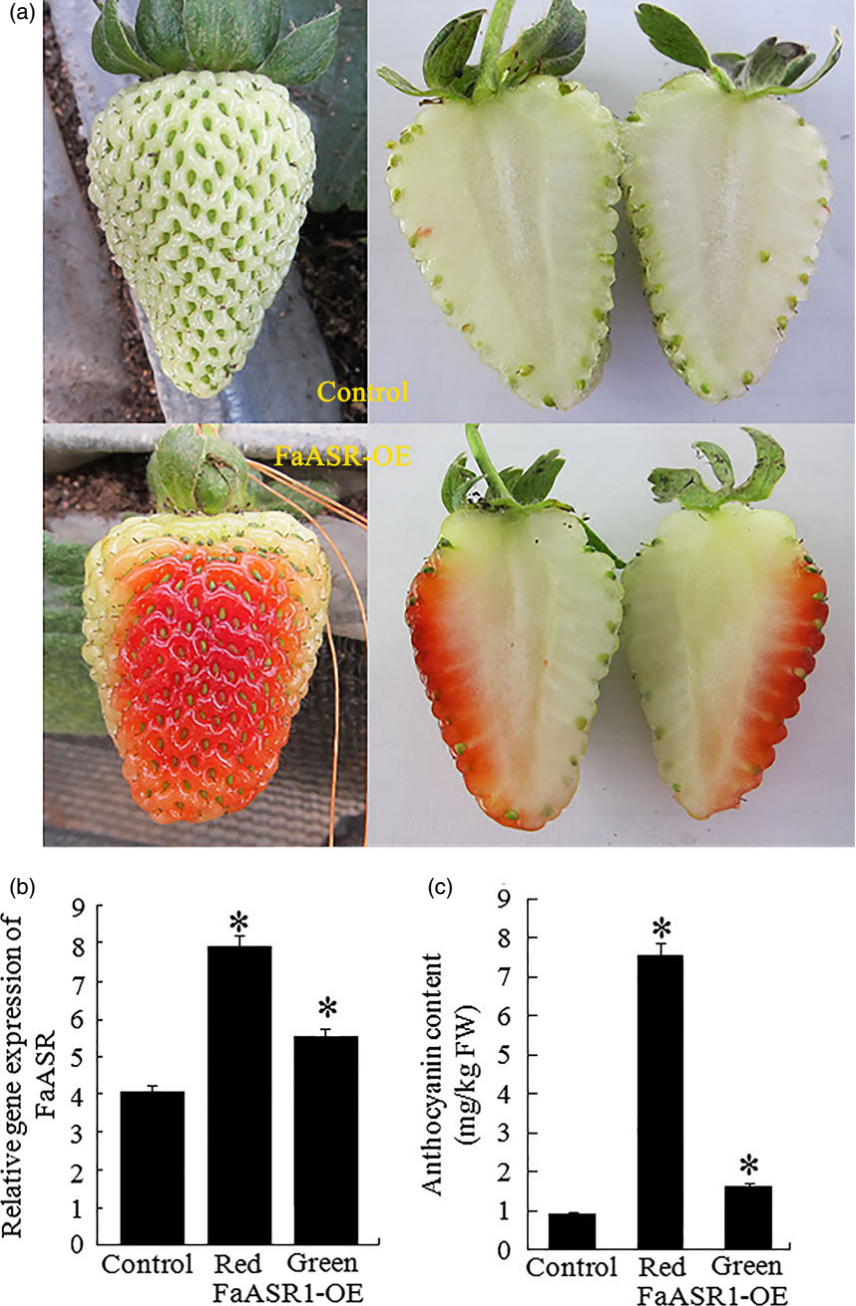
*FaASR* overexpression (OE) accelerated strawberry fruit ripening. (a) *FaASR* expression was performed as described in the materials and methods section. The *FaASR*‐OE construct was generated by cloning the coding region of *FaASR* into the pBI121 vector and placing it under the control of the cauliflower mosaic virus (CaMV) 35S promoter. The *FaASR* construct was injected into the fruits of 12 days after flowering, and empty pBI121 was used as a control. Five days after, injection was indicated. (b) qRT‐PCR analysis of *FaASR* expression in the *FaASR*‐OE and control fruits. (c): control fruit; R: ripening sector of *FaASR*‐OE fruit; G: green sector of *FaASR*‐OE fruit. *FaActin* gene was used as the internal control. Vertical bars represented standard deviations (SD) of means (*n* = 3). (c) The total anthocyanin content was measured in the control and *FaASR*‐OE fruits. Asterisks indicated statistically significant differences at *P* < 0.05 as determined by Student's *t*‐test.

Thirty fruits attached to the plants were injected with the *SlASR1*‐OE (*SlASR1* overexpression) vector, unexpectedly, more than 90% of *SlASR1*‐OE fruits developed a full red colour 8 days after injection, whereas the surface of the control fruit remained without colour red or was partly red (Figure 10a). Real‐time PCR analysis indicated that the mRNA level of the *SlASR1* gene was up‐regulated by 1.5‐fold in the *SlASR1*‐OE fruit compared to that in the control fruit (Figure 10b), and anthocyanin content increased in the *SlASR1*‐OE fruit (Figure 10c). These results indicated that overexpression of the *SlASR1* gene promoted tomato fruit colouring and ripening process. On the other hand, 30 strawberry fruits were injected with the *FaASR*‐OE (*FaASR* overexpression) vector. *FaASR*‐OE fruit turned full red 5 days after injection (Figure 11a) and more than 90% of the analysed fruits appeared to have a similar phenotype; however, the control fruit was partly red. The transcription level of the *FaASR* gene was up‐regulated by twofold in the *FaASR*‐OE fruit compared to that in the control fruit (Figure 11b), and anthocyanin content increased by about sixfold in the *FaASR*‐OE fruit compared to that of the control (Figure 11c), which suggested that the *FaASR* gene promoted fruit colouring in strawberry.

### Alteration of *ASR* gene expression affects a set of ABA‐responsive and ripening‐related gene transcripts levels

**Figure 12:**
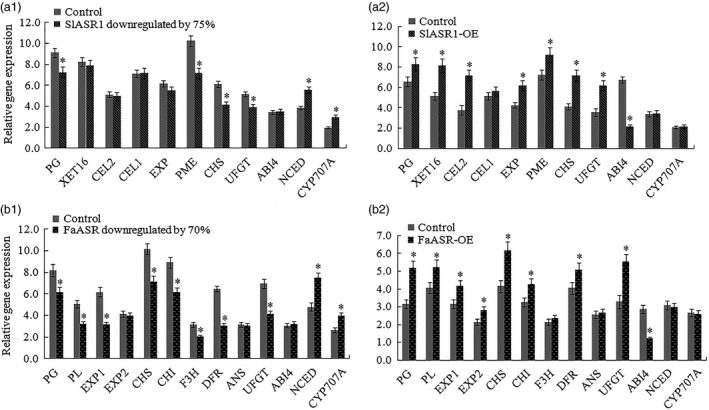
qRT‐PCR analysis of anthocyanin and cell wall metabolism genes. (a) Detection of anthocyanin and cell wall metabolism genes expression levels in the *SlASR1* silencing (a1) or overexpression (a2) tomato fruit and their corresponding control. *SlSAND* gene was used as an internal control. (b) In the strawberry, the cell wall metabolism genes and anthocyanin metabolism genes were analysed in the *FaASR* gene silencing (b1) and overexpression (b2) fruits, and their corresponding control. *FaActin* gene was used as the internal control. Vertical bars represented standard deviations (SD) of means (*n* = 3). Asterisks indicated statistically significant differences at *P* < 0.05 as determined by Student's *t*‐test.

**Figure 13:**
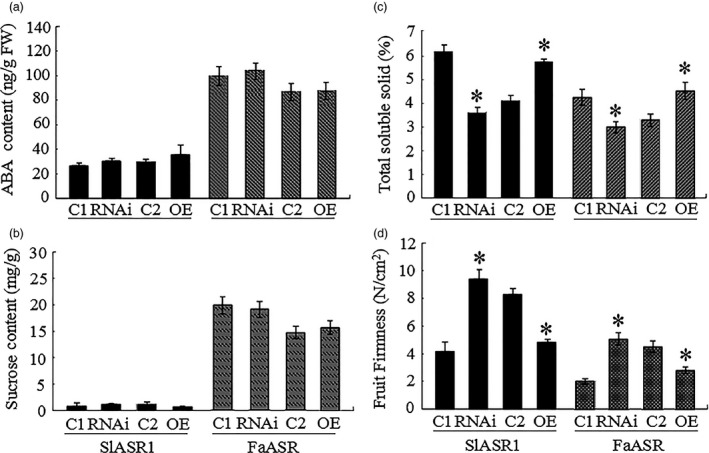
*SlASR1* and *FaASR* gene expression level changes in tomato and strawberry fruit alter fruit physiological parameter, respectively. The physiological parameter of abscisic acid (ABA) content (a), sucrose content (b), total soluble solid content (c) and fruit firmness (d) were analysed in the RNAi and OE of *ASR* gene in tomato and strawberry fruits. The black columns represented the tomato fruit, and other colour columns were strawberry fruit. C1: control of *ASR*‐RNAi fruit; C2: control of *ASR‐* OE fruit. Values were means + SD of four biological replicates. Asterisks indicated statistically significant differences at *P* < 0.05 as determined by Student's *t*‐test.

To elucidate the mechanism of action of the *SlASR1* gene in the regulation of tomato fruit ripening, several ABA‐responsive and ripening‐related genes were examined using both RNAi and OE of the tomato fruit, including transcription factors of the ABA signal transduction pathway, such as the ABA insensitive factor (*ABI4)*, the ABA biosynthesis gene, *NCED*, the ABA degradation gene, *CYP707A*, anthocyanin biosynthesis‐related genes chalcone synthase (*CHS*), chalcone isomerase (*CHI*), anthocyanidin synthase (*ANS*), dihydroflavonol 4‐reductase (*DFR*), flavanone‐3‐hydroxylase (*F3H*), and uridine diphosphate glucose‐flavonoid glucosyltransferase (*UFGT*), and cell wall metabolism‐related genes, polygalacturonase (*PG1*), pectate lyase (*PL*), pectin methylesterase (*PME*), cellulase (*Cel1/2*), xyloglucan endotransglycosylase (*XET16*) and expansin protein (*EXP*). The results showed that in the tomato *SlASR1*‐RNAi fruit, *SlABI4*,* SlCel1*,* SlCel2*,* SlXET16* and *SlEXP1* had no significant differences compared to that of the control fruit, whereas the cell wall metabolism‐related genes, *SlPG* and *SlPME*, and the anthocyanin biosynthesis‐related genes, *SlCHS* and *SlUFGT* were down‐regulated, and the ABA biosynthesis gene, *NCED*, and ABA degradation gene, *CYP707A,* were all up‐regulated (Figure 12a1). On the other hand, in the tomato *SlASR1*‐OE fruit, anthocyanin biosynthesis‐related genes, *SlCHS* and *SlUFGT*, and cell wall metabolism‐related genes, *SlPG1*,* SlPME*,* SlCel1*,* SlXET16* and *SlEXP1,* were all up‐regulated, and the ABA signal transduction pathway *SlABI4* was down‐regulated, but the ABA biosynthesis gene, *NCED*, and ABA degradation gene, *CYP707A*, and cell wall metabolism‐related gene, *SlCel2*, did not significantly differ between the *SlASR1*‐OE and control fruits (Figure 12a2). In strawberry, the ripening‐related genes of the *FaASR*‐RNAi fruit were also analysed at the molecular level. The results showed that the levels of expression of the *FaEXP2*,* FaANS* and *FaABI4* genes did not significantly differ between the *FaASR*‐RNAi and control fruits, whereas the *FaPG*,* FaPL*,* FaEXP1*,* FaCHS*,* FaCHI*,* FaF3H*,* FaDFR* and *FaUFGT* genes were down‐regulated (Figure 12b1), and the *FaNCED* and *FaCYP707A* genes were up‐regulated. On the other hand, in the *FaASR*‐OE fruit, the *FaPG*,* FaPL*,* FaEXP1*,* FaEXP2*,* FaCHS*,* FaCHI*,* FaANS* and *FaUFGT* genes were up‐regulated, and *FaABI4* was down‐regulated. However, no significant difference in the level of expression of the *FaDFR*,* FaF3H*,* FaNCED* and *FaCYP707A* genes between the *FaASR*‐OE and the control fruits was observed (Figure 13b2). These changes in the expression of anthocyanin and cell wall‐associated genes in tomato and strawberry fruit led to fruit colouring and softening. To further analyse the function of the *ASR* gene, the *ASR*‐RNAi fruits were treated with ABA and sucrose, or ABA + sucrose, which resulted in the up‐regulation of the *ASR* gene in strawberry and tomato (Figure S12a, b). In strawberry, anthocyanin biosynthesis‐related genes, *FaCHS*,* FaCHI*,* FaF3H*,* FaDFR* and *FaUFGT*, and cell wall metabolism‐related gene, *FaPG*, were down‐regulated in the *FaASR*‐RNAi fruit, of which the *FaASR* gene expression was silenced (Figure 12b1). However, compared to the nontreated *FaASR*‐RNAi fruit, these genes were up‐regulated in the *FaASR*‐RNAi fruits that underwent ABA, sucrose or ABA + sucrose treatment, whereas the *FaPL*,* FaEXP1*,* FaEXP2* and *FaANS* genes were not induced (Figure S12a). Similarly, the *SlPG*,* SlPME*,* SlCHS* and *SlUFGT* genes were up‐regulated in the *SlASR1*‐RNAi tomato fruit after ABA, sucrose or ABA + sucrose treatment, and *SlXET16*,* SlCEL1* and *SlCEL2* were only induced by ABA and ABA + sucrose, whereas *SlEXP1* was not (Figure S12b). Importantly, the *ASR* gene also responded to various abiotic stresses such as of NaCl treatment, cold and dehydration, as well as biotic stresses of powdery mildew and grey mould infection in strawberry (Figure S13a) and tomato (Figure S13b). Taken together, the *ASR* gene influenced some anthocyanin and cell wall metabolism‐related genes to regulate fruit colouring and softening and was involved in the response of fruits to stresses, which suggested that the *ASR* gene influenced the expression levels of these genes, thereby further affecting fruit quality and protecting the fruit from external injuries.

### Alteration of *ASR* gene expression affects the fruit physiological changes

**Figure 14:**
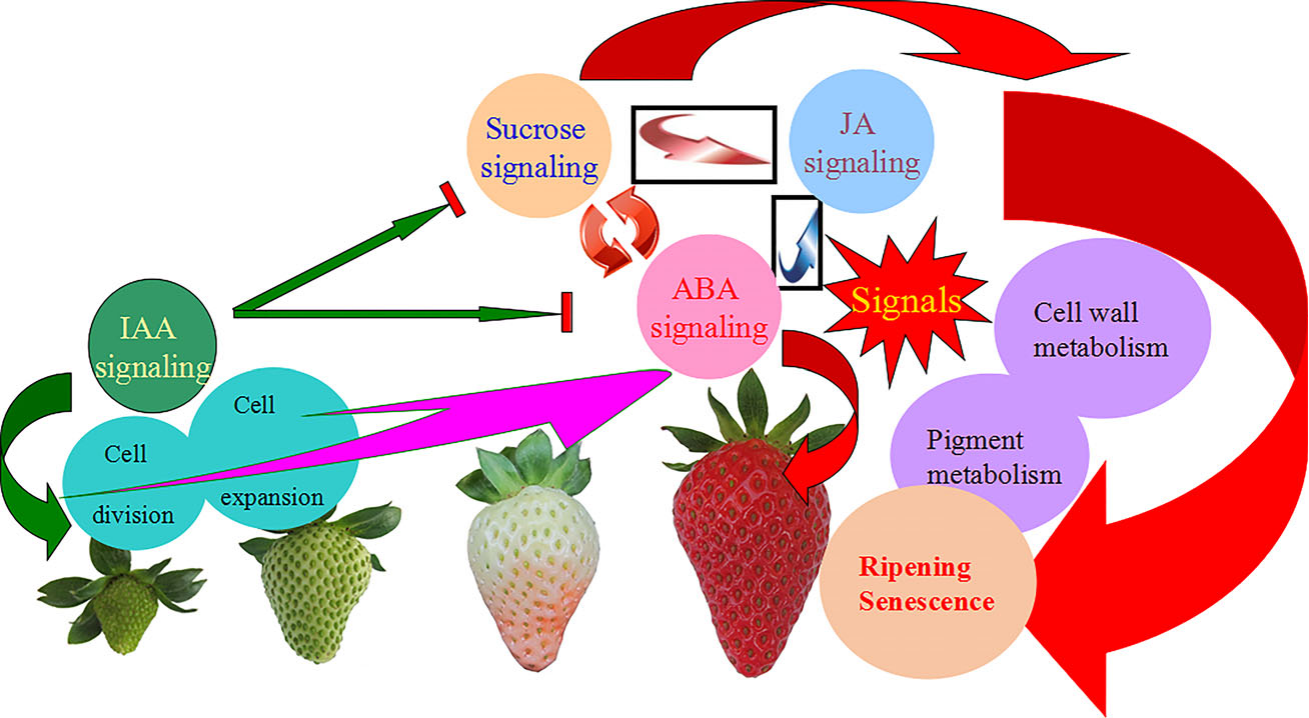
A model of the four signal system relationship: abscisic acid (ABA), IAA, sucrose and jasmonic acid (JA). ABA as the core signal plays an important role the strawberry ripening and senescence, sucrose and ABA can regulate each other, and sucrose is also involved in the fruit ripening. The sucrose and ABA can all induce the JA signal pathway, and JA regulates the fruit ripening through cell wall and anthocyanin metabolism to promote fruit senescence. IAA is a negative factor in the fruit ripening, and it blocks the ABA and sucrose signalling, to delay the fruit ripening and senescence in the later stages of strawberry fruit. In the former stages of strawberry fruit, IAA induces the cell division and expansion. In the same time, the ABA and JA can all inhibit the IAA accumulation to regulate the fruit development.

To determine whether *ASR* gene expression ultimately leads to physiological changes in fruits, some physiological parameters were measured, including fruit firmness, total solid soluble (TSS) content, sugars content and ABA content. Compared to the respective controls, ABA content did not significantly differ between the *SlASR1‐*OE or the *SlASR1‐*RNAi fruit and the relative control fruit of tomato, as well as between the *FaASR‐*OE or the *FaASR‐*RNAi fruit and the relative control fruit of strawberry (Figure 13a). Sucrose content also did not change in tomato *SlASR1‐*OE or *SlASR1‐*RNAi fruits, as well as in strawberry *FaASR‐*OE or *FaASR‐*RNAi fruits (Figure 13b). However, TSS content (Figure 14c) decreased in *ASR*‐RNAi fruits, but increased in the *ASR*‐OE fruits of tomato and strawberry. On the other hand, fruit firmness was greater in *ASR‐*RNAi fruits, but lower in *ASR‐*OE fruits of tomato and strawberry, compared to that of the control fruits (Figure 13d). These data suggested that changes in the expression of the *ASR* gene lead to variations in fruit phenotypes, thereby further affecting fruit quality.

## Discussion

### Physiological changes in strawberry and tomato fruit during ripening

Although the ripening mechanism of tomato and strawberry fruit have several similarities (Jia *et al*., 2011; Sharp *et al*., 2000; Sun *et al*., 2012), these also have differences, such as physiological changes in growth period, development model, fruit firmness and acid (Figures S1 and S2). The present study showed that the differential expression levels of sucrose metabolism genes coincided with varying sucrose levels. Sucrose metabolism is related to four important genes: the sucrose transporter gene (*SUT1*), which transports sucrose from other resources to the fruit (Mahboubi *et al*., 2013); the sucrose phosphate synthase gene (*SPS*), which catalyses the glucose and fructose to form sucrose (Chen *et al*., 2005); the acid invertase gene (*AI*), which degrades the sucrose (Anil *et al*., 1991), and the sucrose synthase (*SS*) gene, which has a dual function that affected sucrose synthesis and degradation (Hou *et al*., 2014). In tomato, the expression level of the *SlSUT* and *SlAI* was higher, but that of the *SlSPS* and *SlSS* genes were lower during fruit development process (Figure S5b), which in turn led to sucrose accumulation, combined with rapid degradation. On the other hand, in strawberry, the high gene expression levels of the *FaSUT* and *FaSPS* genes, and the low gene expression levels of the *FaAI* and *FaSS* genes resulted in a higher rate of sucrose accumulation in strawberry fruit (Figure S5a). Similar to the observed changes due to sucrose, in strawberry, the level of expression of the ABA biosynthesis gene, *NCED*, also consistent with the ABA content during fruit development (Figures S2e and S3b), and ABA activated several ripening‐related genes (Han *et al*., 2015; Jia *et al*., 2011; Li *et al*., 2013; Margherita *et al*., 2013; Molina‐Hidalgo *et al*., 2013; Sun *et al*., 2012). We also determined that the ethylene content peaked in the white strawberry fruit (Figure S2e). Sun *et al*. (2013) recently showed that ethylene promoted strawberry fruit ripening; however, in our study, ethylene did not promote the strawberry fruit ripening, but ABA did (Figure S4). In tomato, the ABA content peak occurred earlier than that of ethylene (Figure S2a), which suggests that ABA activated ethylene accumulation, and ABA and ethylene both promoted tomato fruit maturation. Furthermore, during the later period of tomato development, ethylene played a more prominent role than ABA (Sun *et al*., 2011; Zhang *et al*., 2009).

### Cloning and gene expression patterns of *ASR*

As two main signal systems, ABA and sucrose undergo extensive crosstalk (Carrari *et al*., 2004; Finkelstein and Gibson, 2001). For example, the *ASR* gene could be induced by ABA + sucrose in tomato and strawberry, and a high content of ABA and sucrose remarkably induced ASR gene expression (Chen *et al*., 2011; Figure 6a–e); therefore it is necessary to have a study on the ASR function during fruit development. Presently, ASR have been cloned from various species of dicotyledonous and monocotyledonous plants (Frankel *et al*., 2006), including wheat (Hu *et al*., 2013), maize (Jeanneau *et al*., 2002), ginkgo (Shen *et al*., 2005), banana (Miao *et al*., 2014) and grape (Cakir *et al*., 2003). In grape, the ASR has one ABA/WDS signature, which was described in ABA stress‐ and ripening‐induced proteins (Canel *et al*., 1995) and in water‐deficit stress‐induced proteins (Padmanabhan *et al*., 1997): 5′‐DYRKEEHHKHLEHLGELGVA‐3′ and 5′‐AGAYALHKKHKSEKDPEHAH KHKIEEEIAAAAA‐3′. In addition, the 3′ end of the C‐terminal part of ASR contains a putative signal for nuclear targeting (5′‐KKEAKEEDEEAHGKKHHHLF‐3′). Similar to that in the grape, the ASR in tomato and strawberry also have the His region at its 5′ terminus, ABA/WDS signatures, and one putative signal for nuclear targeting at its 3′ terminus (Figure S6). There are numerous reports on the biochemical and physiological changes of ASR (Dai *et al*., 2011; Golan *et al*., 2014; Henry *et al*., 2011); however, specific findings on the mechanism of the *ASR* gene involved in the fruit ripening are limited. In 2008, Shkolnik and Bar‐Zvi have shown that tomato SlASR1 could abrogate the response to ABA and glucose in *Arabidopsis* by competing with ABI4 for DNA binding; based on the report, we selected *FaASR* and *SlASR1* to research. In present study, the overexpression of the *FaASR* gene in strawberry and the *SlASR1* gene in tomato led to the down‐regulation of the *FaABI4* and *SlABI4* genes, respectively (Figure 12a2,b2), suggesting that the *ASR* gene functioned successfully in strawberry and tomato. A previous report showed that the grape MSA is orthologous to ASR that was bound to the *cis*‐acting element sucrose box, thereby inducing the expression of downstream genes (Cakir *et al*., 2003). In strawberry, three hexose transporter genes, *FaHT1*,* FaHT2*, and *FaHT3*, were all induced by sucrose. Furthermore, its promoters all contained the sucrose box, verified in the tomato *SlHT1* gene and induced by sucrose. The promoter of the *SlHT1* gene had two sucrose boxes (Figure 4a,b). However, one sugar carrier transporter in strawberry was not induced by sucrose (Figure 4a) and had no sucrose‐related *cis*‐element in its promoter. These findings suggested that the sucrose box was specific to sucrose‐inducible genes. The *FaASR* or *SlASR1* gene also induced the activity of the reporter gene, GUS, which was activated by the promoter of the *FaHT* or *SlHT* (Figure 5a–c) and that this *FaASR* or *SlASR1* induction was enhanced by sucrose, ABA or (ABA + sucrose) (Figure 5b), suggesting that the ASR transcription factor that was bound to the hexose transporter promoter activated the expression of the hexose transporter gene and that the ASR responded to sucrose induced the hexose transporter expression was specific. These results further verified that the ASR transcript was induced by sucrose and ABA, which cooperatively regulated the *ASR* transcriptional level (Figure 6a–e), and sucrose‐modulated *ASR* transcriptional level could be mediated via both an ABA‐dependent and ABA‐independent pathway (Figure 7a,b).

### Transcription factor ASR in tomato and strawberry

The four *ASR* genes present in the tomato fruit, *SlASR1*,* SlASR2*,* SlASR3* and *SlASR4*, and one *ASR* gene *FaASR* in strawberry showed different expression patterns in plant tissue (Figure 2a,b) and various fruit developmental process (Figure 3a,b). ASR also responded to various stresses, such as cold, NaCl, dehydration and fungus infection (Figure S13a,b) and thus could play different roles in fruit antistress responses. These findings indicated that ASR members might have different functions in tomato and strawberry growth, as well as respond to different stresses. Taken together, the *ASR* gene positively regulated fruit ripening and stress responses.

To further analyse the *ASR* gene during fruit development, the effect of ABA, IAA, sucrose and JA on the *ASR* gene expression was examined. The results of the present study showed that these were involved in fruit development, and influenced each other during fruit development (Figure S9a–d). ABA and sucrose induced the expression of the *ASR* gene (Figure 6a–e). Furthermore, because sucrose was degraded into glucose and fructose in fruit, we used the sucrose analogue turanose to treat the fruit, and the results showed that turanose also induced *ASR* gene expression (Figure 7a,b), suggesting that sucrose acted on its own action in the fruit and was not due to metabolism. ABA, sucrose, and ABA + sucrose promoted *ASR* gene expression at different induction concentrations and time points (Figure 6a–e). On the other hand, ABA inhibitor, NDGA, did not induce *ASR* expression, whereas sucrose + NDGA did (Figure 7a,b). These findings may be attributable to the fact that the blockage of the ABA signal transduction pathway facilitated in the enhancement of the sucrose signal pathway, which in turn induced *ASR* gene expression. These findings indicate that the influence of sucrose and ABA on *ASR* expression was of two pathways that resulted in the induction of *ASR* expression; furthermore, these two factors may have functioned together or independently to induce ASR gene expression. IAA also inhibited *ASR* gene expression, which was due to the inhibition of IAA on the expression of the *NCED* gene, which in turn promoted the expression of the ABA degradation *CYP707A* gene, thereby leading to the reduction in ABA content (Figure S9c). On the other hand, the inhibition of IAA on the expression of *SUT* and *SPS* genes prevented sucrose accumulation (Figure S9c). These two aspects resulted in a decrease in the level of expression of the *ASR* gene. JA affected the expression of fruit cell wall and anthocyanin metabolism‐related genes (Figure S8), but it had no major influence on *ASR* gene expression (Figure 7a,b). JA has a similar function to that of ABA during plants' response to stress (Anderson *et al*., 2004; Rudell *et al*., 2002), and ABA and JA regulated the same gene expression several times, which suggests that ABA and JA might have engaged in crosstalk. ABA first promoted JA accumulation and then induced the gene expression. Therefore, the genes regulated by JA were also controlled by ABA. In our study, ABA promoted JA accumulation through the induction of JA synthesis gene expression (Figure S9a). However, *ASR* gene expression was not responsive to JA (Figure 7a,b), suggesting that the *ASR* gene was not a downstream gene of JA, and ABA had other pathways to induce gene expression in addition to promoting JA to induce gene expression. Ethylene is a positive regulator of tomato ripening (Sun *et al*., 2010); however, it blocked *ASR* gene expression (Figure 7a,b). ABA activated ethylene production, thereby resulting in cell senescence, although ethylene suppresses anthocyanin accumulation (Jeong *et al*., 2010). Therefore, the role of ethylene in promoting fruit ripening was not due to the activation of fruit colouring, but the induction of cell wall metabolism, which led to substance transformation in the cell, and pigment metabolism increased, ultimately leading to anthocyanin accumulation. The *ASR* gene was related to fruit softening and colouring and had an antistress function during fruit development. Ethylene was a negative regulator of ASR, which led to fruit softening and rapid infection by bacterium. Therefore, ASR and ethylene played different roles in promoting fruit ripening. Taken together, sucrose and ABA both induced the expression of the *ASR* gene. The induction of the expression of the *ASR* gene by sucrose involved two routes: one was dependent on ABA, and the other was independent of ABA. The roles of sucrose on *ASR* gene expression were also independent of IAA. Sucrose, ABA and JA played a positive role in fruit development and ripening, and IAA was negatively involved in fruit ripening and formed a network in regulating fruit development. Furthermore, ASR was involved in the network and played an important role in fruit development.

### Regulation of *ASR* gene expression in tomato and strawberry fruit

To determine the role of the transcription factor ASR in tomato and strawberry fruit ripening, we regulated the level of expression of the endogenous *ASR* gene to analyse the fruit‐ripening process (Figure 8, 9, 10, 11). We found that down‐regulation of the *ASR* gene influenced total soluble solid content (Figure 13c), fruit firmness (Figure 13d) and anthocyanin synthesis (Figures 8c and 9c), as well as delayed ripening‐related gene expression influenced fruit development where the fruit remained firm with no pigment forming (Figure 12a1,b1; 13d; 14). On the other hand, in the *ASR* overexpression fruit, total soluble solid content (Figure 13c) and, anthocyanin accumulation increased (Figures 10c and 11c), the fruit itself was softer (Figure 13d), and the genes associated with cell wall and anthocyanin metabolism were up‐regulated (Figure 12a2, b2). These activities led to a change in fruit phenotype changed (Figures 10a and 11a), and the fruit‐ripening process was accelerated, suggesting that the transcription factor ASR played roles in fruit softening and colouration and was involved in the regulation of fruit flavour and quality. To verify whether the *ASR* gene served as a switch that influenced these genes, ABA, sucrose and ABA + sucrose were used to treat *ASR*‐RNAi tomato and strawberry fruits. The results showed that the *SlASR1* and *FaASR* genes were induced by ABA and sucrose, especially by ABA + sucrose, and the anthocyanin and cell wall metabolism‐associated genes were also induced (Figure S12a,b), indicating that ABA and sucrose influenced tomato and strawberry fruit development by controlling *ASR* gene expression, which in turn that regulated the expression of its downstream genes. In addition, the change of *ASR* gene expression levels was associated with ABA and sucrose signal strength (Figure 6a–e).

### The involvement of hormones and sucrose in fruit ripening

ABA content peaked during tomato fruit development, declined during fruit ripening, induced ethylene production and promoted fruit maturity (Zhang *et al*., 2009; Figure S2a). However, ABA was not the main factor in tomato fruit ripening, with the ethylene signal transduction pathway playing a more important role in tomato fruit ripening (Sun *et al*., 2010). In strawberry, ethylene had no significant effect on the strawberry fruit‐ripening process (Figure S4). ABA content continuously increased from the SG to the FR stages (Figure S2e), and strawberry fruit ripening was mainly regulated by ABA (Jia *et al*., 2011). Therefore, ABA was considered as the regulator of tomato and strawberry fruit maturity. JA also regulates fruit maturity (Mukkun and Singh, 2009; Figure S8). IAA negatively regulates the fruit‐ripening process (Manning, 1994). Sucrose is involved in strawberry fruit ripening (Jia *et al*., 2013), and sucrose and ABA coregulate fruit ripening. Our study presents evidence for a function of an ASR protein acting as a downstream component of a common transduction pathway for ABA and sucrose signals during fruit ripening. Therefore, the relationship of these hormones and sucrose has revealed the ripening mechanism of tomato and strawberry fruits.

As a core signal, ABA influenced the levels of JA, sucrose and auxin (Figure S9a). Auxin promoted fruit expansion and cell division, thereby leading to fruit growth (Table S4). During the early development of the strawberry fruit, IAA levels were high; however, at this same period, low ABA levels existed to keep fruit growth. In the later period of fruit development, a higher level of ABA is produced, and several ripening‐related genes were induced by ABA. It is known that auxin can assist ABA accumulation (Archbold and Dennis, 1984; Manning, 1994; Zhong *et al*., 2004). When the strawberry fruit at the later development period was treated with auxin, the expression of ABA biosynthesis genes was affected and ABA content declined (Figure S9c), which led to the down‐regulation of ripening‐related genes reduced, and thereafter, fruit ripening was delayed. Therefore, ABA and IAA have antagonistic effects on fruit development. Auxin was also influenced by JA (Figure S9d), which had a function of stimulating defensive responses to herbivore and fungus (Avanci *et al*., 2010; Ashish *et al*., 2015) and promoted anthocyanin and cell wall metabolism to induce fruit ripening (Figure S8). JA was also involved in the fruit‐ripening process. JA was downstream of ABA, and its content was influenced by ABA. High levels of ABA induced several ripening‐related genes through JA, but not all the ripening‐related genes responded to JA. JA specifically regulated the mature gene expression (Fan *et al*., 1998; Peña‐Cortés *et al*., 2004). JA could be induced by sucrose (Figure S9b), and a high level of sucrose that produced pigment and aromatic substances in the later stages of fruit development was necessary for fruit maturity. Sucrose is the main source of anthocyanin synthesis, and sucrose accumulation results in fruit flavour and quality improvement (Osorio *et al*., 2013). Sucrose is the product of photosynthesis and is transported from the leaf to the fruit and thus plays a very important role in fruit ripening (Damon *et al*., 1988). In addition to serving as a carbon resource, sucrose also acts as a signal that induces fruit ripening and ABA accumulation (Jia *et al*., 2013). Sucrose signal function was dependent or independent of ABA for the promotion of fruit ripening. Taken together, the ABA was the core signal in the regulation of the development of nonclimacteric fruit strawberry, and sucrose, JA and IAA played different roles in strawberry fruit ripening around ABA (Figure 14).

## Experimental procedures

### Plant material and growth conditions

Octaploid strawberry (*Fragaria *×* ananassa* ‘Fugilia’) plants were grown in a greenhouse (20 °C–25 °C, relative humidity of 70%–85%, 14‐h/10‐h light/dark cycles) during spring seasons from 2014 to 2015. Three hundred flowers on 40 strawberry plants were tagged during anthesis. Fruits at seven growing stages [SG (Small green), LG (Large green), DG (De greening), WT (White), IR (Initial red), PR (Partial red) and FR (Full red)] were collected at 7, 13, 16, 19, 22, 25 and 28 days after anthesis, respectively. Twenty uniformly sized fruits were sampled at every stage (one replicate). After removing the achenes (seeds frozen in liquid nitrogen and stored at –80 °C), the receptacle (pulp) was cut into 0.5–0.8 cm^3^ cubes and was quickly stored at –80 °C after being snap frozen in liquid nitrogen.

Tomato plants (*Lycopersicon esculentum* cv. Ailsa Craig) were grown in greenhouse (20 °C–25 °C, relative humidity of 70%, 14‐h/10‐h light/dark cycles) during 2014–2015. Two hundred flowers on 40 tomato plants were tagged during anthesis. Fruit at eight growing stages [SIG (small immature green), MIG (middle immature green), BIG (big immature green), MG (mature green), B (breaking), T (turning), MR (mature red) and OR (over red)] were collected at 20, 25, 29, 33, 37, 42, 45 and 48 days after anthesis, respectively. Fifteen uniformly sized fruits were sampled at every stage (one replicate). The receptacles (pulp) were cut into 0.5–0.8 cm^3^ cubes and were immediately stored at –80 °C after being snap frozen in liquid nitrogen.

### Cloning of *ASR* genes and bio‐information analysis

The cDNA obtained fellow was used as a template for amplifying the *ASR* genes with primers as described in Table S1. PCR was performed under the following conditions: 94 °C for 5 min, followed by 35 cycles of 94 °C for 30 s, 54 °C for 30 s and 72 °C for 1 min, with a final extension of 72 °C for an additional 10 min. The PCR products were linked into a pMD‐T simple vector (TaKaRa, Kusatsu, Shiga, Japan) and subsequently transformed into *Escherichia coli* DH5a. Positive colonies were selected, amplified and sequenced by Invitrogen China (Shanghai, China). Multiple sequence alignment of ASRs was performed using the ClustaX program (version1.81) and shaded with the website http://sourceforge.net/projects/boxshade/. Phylogenetic tree were constructed using MEGA 4.1 program with the neighbour‐joining (NJ) method and bootstrap analysis (1000 replicates) (Saitou and Nei, 1987). The ASR protein used for phylogenetic tree as described in Table S2.

### RNA isolation and RT‐PCR Analysis

Total RNA was extracted from 10 g of fresh or treated tomato and strawberry fruits using an RNA extraction kit (SV Total RNA Isolation System; Promega, Madison, Wisconsin America BioTeke, Beijing, China). Genomic DNA was eliminated with a 15‐min incubation at 37 °C with RNase‐Free DNase (TaKaRa Bio), followed by the use of an RNA Clean Purification Kit (BioTeke). The purity and integrity of the RNA were determined by agarose gel electrophoresis and the A260‐A230 and A260‐A280 ratios (a ratio of about 2.0 is generally accepted as ‘pure’ for RNA). The DNA contamination detection was performed by PCR; for tomato, the PCR primer was for *SlSAND* gene fragment containing one intron (NCBI Reference Sequence: NC_015440.2; forward: 5′‐TTTGATGGAGGTTCGAACTC‐3′; reverse: 5′‐TGACTCAAGACAAAGAAGTG‐3′); for strawberry, the PCR primer was for *FaActin* gene fragment containing one intron (NCBI Reference Sequence: NC_020496.1; forward: 5′‐CTGTTCTTTCCCTCTATGCT‐3′; reverse: 5′‐TCAGCTGTGGTGGTAAATGA‐3′). To generate first‐strand cDNA, 3 μg of total RNA was reverse transcribed using a Clontech kit (TaKaRa Bio) according to the manufacturer's protocol. Random primers were used to reverse transcribed the first‐strand cDNA of strawberry fruit and tissues. The real‐time PCR primers for genes were designed as in Table S5.

Real‐time RT‐PCRs (20 μL) contained 10 μL SYBR Premix Ex Taq (perfect real‐time buffer contained dNTPs, MgCl_2_ and DNA polymerase; TaKaRa), 0.4 μL 10 μm forward specific primer, 0.4 μL 10 μm reverse specific rimer (Invitrogen) and 2 μL cDNA template. The mixture was placed in a Bio‐Rad iQ5 Sequence Detector (Bio‐Rad, Hercules, CA), and DNA amplification was conducted using the following thermocycling programme: 1 cycle of 95 °C for 2 min 40 cycles of template denaturation at 94 °C for 20 s, primer annealing at 56 °C for 20 s, primer extension at 72 °C for 30 s and 71 cycles increasing from 60 °C to 95 °C at 0.5 °C per cycle for 30 s. The sequence detector was programmed to measure fluorescence only during the annealing step. At this temperature, no incorporated uniprimer was in the hairpin conformation contributing to fluorescence measurements. Normalization of the expression of other genes according to the reference genes of *SlSAND* gene (Accession No: SGN‐U316474) for tomato and *FaActin* gene (AB116565) for strawberry, respectively. Relative fold expression changes were calculated using the relative two standard curves method with Rotor‐Gene 6.1.81 software (Invitrogen).

### Construction of the expression vector and *Agrobacterium*‐mediated infiltration

The promoter sequences of HT in fruit were isolated according to information provided at strawberry library (https://strawberry.plantandfood.co.nz/index.html) and tomato gene library (http://solgenomics.net/). The primers used were as described by Table S1. These products were linked into the PMD20‐T vector and subsequently transformed into *E. coli* DH5α. Positive colonies were selected and amplified and then sequenced by Invitrogen. Promoter analysis was performed using PLACE (http://www.dna.affrc.go.jp/PLACE/signalscan.html; Higo *et al*., 1999). For the promoter of *HT* vector construction, the 35 s promoter of binary expression vector pBI121 was cut with restriction site and was replaced with the *HT* promoter from strawberry and tomato, respectively. These promoters used were 3000 bp length from the transcription site of *HT* gene.

For over expression of tomato *ASR* gene, the tomato *SlASR1* gene sequence was selected and amplified using appropriate primers as described in Table S1 and then cloned the sequence into the pMD‐T simple vector (TaKaRa), digested with XbaI and SacI for tomato and for strawberry, and subsequently cloned into the binary expression vector pBI121 cut with the same restriction enzymes. pBI121 or the pBI121 derivative, pBI121‐*SlASR*‐*330* and pBI121‐*FaASR*‐*579,* was transformed into *Agrobacterium* strain GV3101 by the freeze–thaw method. For RNAi of tomato and strawberry *ASR* gene, the primers were used as the same with the primers of the overexpression. They were cloned into the pMD‐T simple vector (TaKaRa), digested with XbaI and SacI for tomato and for strawberry, and subsequently cloned into the tobacco rattle virus TRV2 cut with the same restriction enzymes. TRV1, TRV2 or the TRV2 derivative, TRV2‐*SlASR1*‐*330* and TRV2‐*FaASR*‐*579,* were transformed into *Agrobacterium* strain GV3101 by the freeze–thaw method (Figure S10, Fu *et al*., 2005). About 5 mL culture of each strain was grown overnight at 28 °C in Luria–Bertani (LB) medium (50 mg/mL kanamycin and 50 mg/mL rifampicin, 10 mm MES, 20 μm acetosyringone). The overnight cultures were inoculated into 50 mL of LB medium and grown at 28 °C overnight. The cells were harvested by centrifugation (3000 ***g***, 5 min, 20 °C) and suspended in infiltration buffer (10 mm MgCl_2_, 10 mm MES, 20 μm acetosyringone), adjusted to an optical density (OD) of 0.8–1.0 of pBI121 and its derivatives, and for TRV1 or TRV2 and its derivatives, the OD_600_ were 1.0–2.0, and left to stand at room temperature for 4 h. About 1 mL of *Agrobacterium* was infiltrated into every LG fruit with a 1‐ml syringe. Ten uniformly sized fruit were used in infiltration experiment, and the experiment was repeated three times.

### Incubation of fruit disc tissue *in vitro*

Treatment on tissues of strawberry fruit with ABA, IAA, MeJA, NDGA, NDGA + sucrose, sucrose, sucrose analogue turanose and ethephon by an *in vitro* incubation was performed as described by Beruter and Studer (1995). After being washed by distilled water, the freshly harvested berries were pre‐cooled to 4 °C. Discs of berry, 10 mm in diameter and 1 mm in thickness, were prepared with a cork borer. The discs were immediately immersed in the equilibration buffer for 30 min with 200 mm mannitol (Archbold, 1988). The equilibration buffer consisted of 50 mm MES (pH 5.5), 10 mm MgCl_2_, 10 mm EDTA, 5 mm CaCl_2_ and 5 mm Vc. The discs were divided into eight sections, and one section was incubated in equilibration buffer with 200 mm mannitol as control, and the others were incubated in equilibration buffer with 50 μm of ABA, IAA and JA, 100 μm of NDGA and NDGA(100 μm) + sucrose(100 mm), and 100 mm of sucrose or turanose, and 100 μm ethylene, respectively. As to the buffer, mannitol was used to adjust the incubation system and make it equal in osmotic potential. The eight sections were placed in 250 mL erlenmeyer flask and shaked at 25 °C for 8 h. After being washed by double distilled water, the tissues were used immediately for assays or frozen in liquid nitrogen and kept at −80 °C until use.

### Effect of abiotic and biotic stresses on strawberry fruits

The fruits attached to the plants were treated as follows: For NaCl treatment, the concentration of 25 mm NaCl was injected in the strawberry and tomato fruits; For cold treatment, the fruits attached to the plant were put in the refrigeratory house of 4 °C; For dehydration treatment, the root of plant was outside in the dry environment to make the plant loss of water of about 10% and further induced the fruits to lose water. One day later, these treatment fruits were collected, and total RNA was isolated immediately. The grey mould and powdery mildew treatment was that the surface of strawberry and tomato fruits were spray with grey mould and powdery mildew spores when approaching to fruit ripening, and the ripening fruits were collected after fruits infection. Control fruits were used for these treatment was that normal ripening fruit.

### Determination of fruit physiological changes

To know the physiological state of the tomato and strawberry development, fruit size, firmness, total soluble solid (TSS) content, sugars content, anthocyanin contents, hormones ABA, and ethylene were analysed. The fruit size was determined with vernier calipers. The fruit firmness was measured after removal of the skin on three sides using a fruit hardness tester (FHM‐5; Takemura Electric Work Ltd, Toshima‐ku, Tokyo, Japan). The TSS content of flesh was measured using a hand‐held sugar measurement instrument (MASTER‐100H, ATAGO Master, Japan), onto which fruit juice was applied to obtain a reading. Anthocyanin concentration was measured by extracting receptacle surface of equal weight (five replications) with 1% HCl methanol and determining the absorbance at 530 and 657 nm. The formula A = A530–0.25 A657 was used to compensate for the contribution of chlorophyll and its degradation products to the absorption at 530 nm. The anthocyanin concentration was a relative value, and we set A = 0.01 equal to 1 unit (Fuleki and Francis, 1968a,b; Rabino and Mancinelli, 1986). The hormone ABA and anthocyanin content were determined as described by Jia *et al*. (2011). The ethylene was determined as described by Sun *et al*. (2012).

### Tobacco transient expression and GUS activity assays

We used *Agrobacterium*‐mediated tobacco transient expression of proteins for GUS activity analysis. The constructs were infiltrated into 2 weeks old leaves of *N*. *benthamiana*. First, the *Agrobacterium* was resuspended in infection solution (10 mm MgCl_2_, 10 mm 2‐(N‐morpholino) ethanesulfonic acid (pH 5.6), autoclaving for 15 min, 100 μm acetosyringone), and then, the 1‐mL syringe was used to inject the *Agrobacterium* into leaf from leaf epidermal by pressing the syringe with thumb. After injection, the tobacco leaves will appear moist phenomenon. Two to 5 days later, the leaf was collected, or spraying with ABA (60 μm) and sucrose (100 mm) on the leaf to treat for 8 h, and then, the GUS activity was measured (Du *et al*., 2013).

Fluorometric GUS assays were performed based on a method described by Jefferson *et al*. (1987). The leaf tissues of transgenic tobacco plants were grounded in the presence of liquid nitrogen in a mortar, and transferred to a microtube. One millilitre of the extraction buffer [50 mm NaH_2_PO_4_, pH 7.0, 1 mm EDTA, 0.1% Triton X‐100, 0.1% (w/v) sodium lauryl sarcosine, and 5 mm dithiothreitol] was added and mixed. The supernatant, after being centrifuged at 12 000 ***g*** for 10 min at 4 °C, was assayed for GUS activity with 4‐methylumbelliferyl glucuronide (MUG) substrate using a F‐4500 fluorescence spectrophotometer (Hitachi, Tokyo, Japan) at the excitation/emission wavelengths of 365 nm/455 nm, as described by Jefferson *et al*. (1987). The protein concentrations were quantified using bovine serum albumin (BSA) as a standard, according to Bradford (1976) and the GUS enzyme activity, which was expressed as nmol of 4‐methylumbelliferone (MU) produced per mg protein per min.

### Detection of the TRV vector by RT‐PCR

Random primers were used to reverse transcribe RNA for the first strand of infiltrated strawberry fruit to detect the TRV vector. RT‐PCR primers for TRV genes were designed as follows: RNA1 primers (GenBank Accession No. AF406990; forward: 5′‐TTACAGGTTATTT GGGCTAG‐3′, and reverse: 5′‐CCGGGTTCAATTCCTTATC‐3′); and RNA2 primers (GenBank Accession No. AF406991; forward: 5′‐TTACGACGAACCAAGGGAGTACTAC‐3′, and reverse: 5′‐AGTCACAATTAGCCCTATTTAGATGT‐3′). PCR was performed under the following conditions: 94 °C for 5 min, followed by 35 cycles at 94 °C for 30 s, 54 °C for 30 s and 72 °C for 1 min, with a final extension at 72 °C for an additional 10 min.

### siRNA test

Total RNA (50 μg) was isolated as described above, digoxigenin (DIG)‐labelled probes were synthesized using a PCR‐DIG Probe Synthesis Kit (Roche Diagnostics, Basel, Switzerland) according to the manufacturer's protocol. Total RNA was fractionated in a 15% (w/v) polyacrylamide urea gel and blotted onto a 0.45 mm nylon membrane (Whatman, Nytran SPC, Sanford, CA) and hybridized with an *FaASR*‐specific probe corresponding to the *FaASR* region (sense: 5′‐ATGTCTGACGAGAAG CACCACCAC‐3′ and antisense: 5′‐GAAGAGATGATGGTGCTTC‐3′). rRNA stained with ethidium bromide was used as a gel loading control. The filters were hybridized overnight with DIG‐labelled DNA probes (0.3–1.0 g/mL) in high stringency hybridization solution (50% formamide, 2× SSPE buffer, 10 mm dithiothreitol, 1 mg/mL herring sperm DNA, 500 μg/mL yeast tRNA and 1 mg/mL bovine serum) in a shaking water bath at 50 °C. Following hybridization, the filters were washed twice at 50 °C for 15 min in each of 2 × SSC, 1 × SSC and 0.1 × SSC. The membranes were then subjected to immunological detection according to the manufacturer's instructions using NBT/BCIP stock solution as a chemiluminescent substrate for alkaline phosphatase (Roche Diagnostics) (Guo *et al*., 2003).

## Conflict of interest

The authors declared that they have no conflict of interests.

## Supporting information


**Figure S1** Morphological and physiological changes in the receptacle of strawberry fruit and tomato fruit during developmental processes.
**Figure S2** Physiological change in tomato and strawberry fruit.
**Figure S3** qRT‐PCR of *NCEDs* genes expression level during tomato and strawberry fruit development.
**Figure S4** The effects of exogenous ABA (50 μm) and ethephon (50 μm) on strawberry fruit‐ripening process.
**Figure S5** qRT‐PCR of sucrose metabolism‐related genes expression level in the process of tomato and strawberry fruit development.
**Figure S6** Amino acid sequence alignment of tomato SlASR and strawberry FaASR with other plant ASR proteins.
**Figure S7** Determination of *ASR* gene expression level induced by ABA and sucrose in strawberry and tomato.
**Figure S8** The influence of jasmonic acid on the fruit ripening‐related genes.
**Figure S9** Determination of the relationship of four ripening‐related factors: ABA, JA, IAA, and sucrose in strawberry.
**Figure S10** Construction of pTRV1, pTRV2 and pTRV2‐derivative pTRV2‐*SlASR1* or pTRV2‐*FaASR*.
**Figure S11** SqRT‐PCR analysis of TRV expression in fruits.
**Figure S12** Effect of ABA, sucrose, and ABA + sucrose on the cell wall and anthocyanin metabolism gene expression levels in the *ASR*‐RNAi fruit of strawberry and tomato.
**Figure S13** The abiotic and biotic stress on the *ASR* gene expression level of strawberry and tomato fruit.
**Table S1** Specific primers used for amplification genes.
**Table S2** ASR gene sequences used for phylogenetic tree analysis.
**Table S3** Similarity of Sl/FaASRs based on deduced amino acid sequence (%).
**Table S4** The hormone auxin affects the strawberry fruit expansion.
**Table S5** Specific primers used for real‐time PCR analysis.
