# Supplementary material for: Abscisic acid and sucrose regulate tomato and strawberry fruit ripening through the abscisic acid‐stress‐ripening transcription factor
Source: Plant Biotechnol J. 2016 May 4;14(10):2045–65. doi: 10.1111/pbi.12563 (PMC5043491; doi:10.1111/pbi.12563)
Supplement: Supplementary file 1 — Figure S1 Morphological and physiological changes in the receptacle of strawberry fruit and tomato fruit during developmental processes. Figure S2 Physiological change in tomato and strawberry fruit. Figure S3 qRT‐PCR of NCEDs genes expression level during tomato and strawberry fruit development. Figure S4 The effects of exogenous ABA (50 μm) and ethephon (50 μm) on strawberry fruit‐ripening process. Figure S5 qRT‐PCR of sucrose metabolism‐related genes expression level in the process of tomato and strawberry fruit development. Figure S6 Amino acid sequence alignment of tomato SlASR and strawberry FaASR with other plant ASR proteins. Figure S7 Determination of ASR gene expression level induced by ABA and sucrose in strawberry and tomato. Figure S8 The influence of jasmonic acid on the fruit ripening‐related genes. Figure S9 Determination of the relationship of four ripening‐related factors: ABA, JA, IAA, and sucrose in strawberry. Figure S10 Construction of pTRV1, pTRV2 and pTRV2‐derivative pTRV2‐SlASR1 or pTRV2‐FaASR. Figure S11 SqRT‐PCR analysis of TRV expression in fruits. Figure S12 Effect of ABA, sucrose, and ABA + sucrose on the cell wall and anthocyanin metabolism gene expression levels in the ASR‐RNAi fruit of strawberry and tomato. Figure S13 The abiotic and biotic stress on the ASR gene expression level of strawberry and tomato fruit. Table S1 Specific primers used for amplification genes. Table S2 ASR gene sequences used for phylogenetic tree analysis. Table S3 Similarity of Sl/FaASRs based on deduced amino acid sequence (%). Table S4 The hormone auxin affects the strawberry fruit expansion. Table S5 Specific primers used for real‐time PCR analysis. [file PBI-14-2045-s001.docx]

| Supplementary data  Table S1. Specific primers used for amplification genes. | | | |
| --- | --- | --- | --- |
| gene | Forward primers | Reverse primers | Accession |
| SlASR1 | TCGGTAAACTTGGCACTG | TTAGAAGAGATGGTGGTGTC | SL2.31sc04135 http://solgenomics.net/ |
| SlASR2 | CCATCACCATTTGTTCCAC | TCTCTTCCTCTATCTTGTGC | SL2.31sc04135 |
| SlASR3 | ATCACCGTTTGTTCCACC | GTCTTTCTTTGCCTTGTGC | SL2.31sc04135 |
| SlASR4 | CCACCACAAGAACAAAGAG | CCACCTTCCTCATTACCAT | SL2.31sc04135 |
| FaASR | GAAACCTCCTCCTACGAATC | CTCCTCTTCTACCTTGTGC | Gene 08120 locus in https:// strawberry.plantandfood.co.nz/index.html |
| SlASR1-OE | TCTAGAATGGAGGAGGAGAAACACTA | GAGCTCGAAGAGATGGTGGTGTC |  |
| SlASR1-RNAi | GAGCTCATGGAGGAGGAGAAACACTAC | TCTAGATTAGAAGAGATGGTGGTGTC |  |
| FaASR-OE | TCTAGAATGTCTGACGAGAAGCACCACCAC | GAGCTCGAAGAGATGATGGTGCTTC |  |
| FaASR-RNAi | GAGCTCATGTCTGACGAGAAGCACCACCACCAC | TCTAGATAAGAAGAGATGATGGTGCTTC |  |
| Full-length-SlASR1 | ATGGAGGAGGAGAAACACTAC | GAAGAGATGGTGGTGTC |  |
| Full-length-SlASR2 | ATGGCTGAAGAGAAAACAAC | TTAGTAGTGGTGGTGGTGGT |  |
| Full-length-SlASR3 | ATGGCTGAAGAGAAACAAC | TTAGTGGTGTCGTCCTTTC |  |
| Full-length-SlASR4 | ATGGCTGAAGAAAAGAAAC | TTAGAAGAAATGATGCTTC |  |
| Full-length-FaASR | ATGTCTGACGAGAAGCACCACCAC | TAAGAAGAGATGATGGTGCTTC |  |
| FaASR-probe | CCTCCTCCTACGAATCTAAG | CTCCTCTTCTACCTTGTGC |  |
| FaHT1 | CCTTCTTTGCCTCTTCTGTC | TCCACATCCTCCATTCCTC | gene26442 |
| FaHT2 | GCCACAATAGCAACCTTC | GTCTCCTTACCAACATCCC | gene21179 |
| FaHT3 | AGGTGAAGGTGGACAATAC | AGTGCCATAGTTGATGAGG | gene21181 |
| FaSC | CAATGGGTGTGTGGATGT | GGGTTCTCTATCTTCTTGGAC | XM_004295865 |
| SlHT1 | CCATACAAATACAGAGGAGCAC | ACAGCAGACATAAGTGAAGC | NM_001247920 |
| Promoter  FaHT1 | GGGCACATCATTAAGACAGGAAGC | CTTCCAAGCAGAGACACAAGAGC |  |
| Promoter  FaHT2 | TTCTTCATTTTGGTATACCTTTTG | TCTGTTCTTATATGGTCGCCTA |  |
| Promoter  FaHT3 | TGATGAAGAAGATACATAGCATTC | CTCCCACAGTAGTATTAACATGGT |  |
| Promoter  FaSC | CTTCGACCAGCCAAATCTATCTCCTC | CTTTAGATTACAATAAACATCAC |  |
| Promoter  SlHT1 | CCACACCAACAAAATTAAAGCAGTC | ATAAATTTGATAATGAAAGAGAAG |  |

Table S2. ASR gene sequences used for phylogenetic tree analysis.

| Abbreviations | Name | Scientific name | Accession |
| --- | --- | --- | --- |
| CmASR | Pemelo | *Citrus maxima* | U18972 |
| CsASR | Tea | *Camellia sinensis* | KF880380 |
| OsASR | Rice | *Oryza sativa* | KF916487 |
| ZmASR | Ber | *Ziziphus mauritiana* | JN813431 |
| SlASR | Suaedaforsk | *Suaeda liaotungensis* | KC460335 |
| LcASR | Lychess | *Litchi chinensis* | JX291143 |
| MaASR1 | Plantain | *Musa acuminata* | AY628102 |
| ZmASR | Maize | *Zea mays* | EU963502 |
| GmASR | Soybean | *Glycine max* | AY382827.1 |
| PvASR | Bean | *Phaseolus vulgaris* | KF033741 |
| MtASR | Alfalfa | *Medicago truncatula* | XM_003592050 |
| RcASR | Castor | *Ricinus communis* | XM_002524251 |
| PpASR | Peach | *Prunus persica* | AF317062 |
| PmASR1 | Plum | *Prunus mume* | AB434496 |
| PmASR2 | Plum | *Prunus mume* | AB434494 |
| PmASR3 | Plum | *Prunus mume* | AB434495 |
| PaASR | Apricot | *Prunus armeniaca* | U93164 |
| MdASR | Apple | *Malus x domestica* | XM_008383612 |
| VpASR | Grape | *Vitis pseudoreticulata* | DQ336286 |
| PbASR | Pear | *Pyrusx bretschneideri* | XM_009378267 |
| TcASR | Cocao | *Theobroma cacao* | XM_007019478 |
| PeASR | Populus | *Populus euphratica* | XM_011045938 |
| VvASR | Grape | *Vitis vinifera* | AF281656 |
| CmASR | Muskmelon | *Cucumis melo* | AF426403 |
| PtASR | Loblolly | *Pinus taeda* | U52865 |
| NnASR | Lotus | *Nelumbo nucifera* | XP_010260981 |
| MaASR2 | Plantain | *Musa acuminata* | XP_009393803 |
| EgASR | Eucalypt | *Eucalyptus grandis* | XP_010062105 |
| MbASR | Plantian | *Musa balbisiana* | ACZ50740 |
| GbASR | Gingko | *Ginkgo biloba* | AAR23420 |

Table S3. Similarity of Sl/FaASRs based on deduced amino acid sequence (%).

| Gene name | SlASR1 | SlASR2 | SlASR3 | SlASR4 | FaASR |
| --- | --- | --- | --- | --- | --- |
| SlASR1 | 100 | 82.6 | 75.5 | 73.6 | 72.7 |
| SlASR2 |  | 100 | 84.3 | 75.9 | 71.2 |
| SlASR3 |  |  | 100 | 73.1 | 65.7 |
| SlASR4 |  |  |  | 100 | 52.6 |
| FaASR |  |  |  |  | 100 |

Table S4. The hormone auxin affect the strawberry fruit expansion. 50 µM IAA was use to even spread the strawberry, the water was used to as the control. 90 fruits that is 7 days after flowering were selected and treated. Seven days later their fresh weight, horizontal and vertical diameter were determined. They acted as the parameter to analyze the IAA on the fruit expansion. * and ** indicated the number of water and IAA treated fruits, respectively. For water treatment, the fresh weight of 33 fruits was below 2.49g, 9 fruits was above 2.49g, but for IAA treatment, 8 fruits was below 2.49g, and 40 fruits was above 2.49g.

| Fruit number  (Water) * | | Fruit number (50 µM IAA)  ** | | Fresh weight (g) | Horizontal  diameter (cm) | Vertical diameter (cm) |
| --- | --- | --- | --- | --- | --- | --- |
| Before treatment | | Before treatment | | =0.95±0.05 | =0.6±0.02 | =0.7±0.02 |
| After treatment | *  33 | After treatment | **  8 | ≦2.49 g | ≦1.1 | ≦1.3 |
| After treatment | *  9 | After treatment | ** 40 | >2.49 g | >1.1 | >1.3 |

| Table S5. Specific primers used for real-time PCR analysis. | | | |
| --- | --- | --- | --- |
| gene name | Forward primers | Reverse primers | Accession |
| SlASR1 | GTTCCACCACAAGGACAA | ACAGTGCCAAGTTTACCG |  |
| SlASR2 | CCATCACCATTTGTTCCAC | CACCAAGTTCACCAATCTTC |  |
| SlASR3 | GAAGCACCATAGCCATCT | TCTGGGTCTTTCTTTGCC |  |
| SlASR4 | AATGAGGAAGGTGGCTATG | CTATCTTGTGCTTGTGTGC |  |
| FaASR | CGAGACCTCCACTGAAACT | TACCTTGTGCTTGTGTGC |  |
| FaNCED1 | TGCTTATCTCGCCATTGC | CGAAGGAAGGAAGAAAGGC | JX013944 |
| FaNCED2 | AACGGTTCGTCGTAATCC | ATGGAAGCAGAAGCAGTC | JX013945 |
| FaNCED3 | CATTGCGGGTTGAACTATCC | GTTTCCAAGTCCACCTTAGC | JX013946 |
| SlNCED1 | AGGCAACAGTGAAACTTCCATCAAG | TCCATTAAAGAGGATATTACCGGGGAC | Z97215 |
| SlNCED2 | TGGTTTTCATGGGACATTCATTAGC | ATCTCCCTTCTCAACTCCCTATTCC | EU912387 |
| SlPG | AAGCATGGAATGAAGCATGTTCATCTAG | CAAAAGCAATCCAAAGCCTTCTATC | X05656 |
| SlXET16 | GTGGGATGAACCTGTTATGTCCGAG | TATGATTCGTCTTGTGCTGGTGGTG | AK246244 |
| SlCel1 | AGTTGCCTCTGAGTTTAGTTGGGATG | TCCACCTGGGGTTGTCTTAATTTGTA | U13054 |
| SlCel2 | CCACAAAGGATTCACCATAGGGGTAG | ATTCTTGAAAATAGGGTCTGGCGTCT | U13055 |
| SlEXP1 | AATCAAATGCGGTTTTAACTGGTCAAT | TCGATTTCTTTTCCTAAGGTGAACAAC | U82123 |
| SlPME | CAGAGCTGGCATCTTGATGGCTACT | GCAATGTATATCGCGGTCCATGACT | Z94058 |
| SlCHS | GAGTCCTTGTTGTTTGCTC | CTGCTGAGACGAGTTCAA | NM_001247104 |
| SlUFGT | CCAACAAGTTACAGCGAC | CAATGGGACACAAATCCTC | XM_004247965 |
| SlABI4 | AGAGGTGTAAGGAAGAGGC | TTCGCTTTAGGTCCACGA | XM_004243323 |
| FaPG | CGACAGAGTGAAAAATTCCTTAG | AGGACTGGGTTAGCAAAATTATTC | EF441274 |
| FaPL | TGACTCCCTTGCTGCTTCTT. | TCTACTGCGTGCTCATTCCA | EF441273 |
| FaEXP1 | GCACTGCCGGAAAGCCCTCCATT | TCCGGCTTTGTACTCGGCGATCTTG | [AF163812](http://www.ncbi.nlm.nih.gov/nucleotide/6715548?report=genbank&log$=nucltop&blast_rank=1&RID=DGHR8F0P015) |
| FaEXP2 | CTTCTCCTTCTAGCTAGC | GCATGGCCACCAACCCAA | [AF159563](http://www.ncbi.nlm.nih.gov/nucleotide/6646884?report=genbank&log$=nucltop&blast_rank=2&RID=DGHRNBBA014) |
| FaCHS | GCTGTCAAGGCCATTAAGGA | GAGCAAACAACGAGAACACG | [XM_004306495](http://www.ncbi.nlm.nih.gov/nucleotide/470141649?report=genbank&log$=nucltop&blast_rank=1&RID=DGHPGF7W014) |
| FaCHI | GTTAAGTGGAAGGGCAAGA | CCCGTCAGCGGTAGTATCA | AB201755 |
| FaF3H | TTTTCTGAGCAATGGGAGG | CTGGGTTCTGGAATGTCG | AB201760 |
| FaDFR | ACGAAGTGATAAAGCCAACA | AAACACCAACCTCCGAAC | AF029685 |
| FaANS | CGTGAGACCCAAAGAGGA | ATGCCGTGGTTGATAAGG | AY695818 |
| FaUFGT | GGTAAGCCACAGGAGGACA | TATGAGCACCGAACCAAAA | AY575056 |
| FaABI4 | GTCGTCTTCTTCTTCCACC | CGGAGGAGTTAGGGTAAAC | XM_004308898 |
| FaBG3  FaBG3 | ACGCAGGGGAGCTAGTTAAAGTCGT | AAACTCATCAACGCCATTCTCTGTG | JX244263 |
| FaPYR1 | TCGTAATGAGCGTGGGAT | GATTCCAGAACAACTGTCCA | JF268669 |
| FaCYP707 | AGGTGTTCTCTTTGCTGC | ATGTTTCTGGTGTCTGCC | HQ681284 |
| FaAOS | CAGAACCCTCTCCTATCTCG | GATTGTGACACGCTTCGTC | [XM_004291875](http://www.ncbi.nlm.nih.gov/nucleotide/470111373?report=genbank&log$=nucltop&blast_rank=1&RID=DH1E39FK014) |
| FaOPDA1 | GAAGCCACTGGAGTTTCTG | AGAATACACCACCTTTAGCAT | \| [XM_011102912](http://www.ncbi.nlm.nih.gov/nucleotide/747105872?report=genbank&log$=nucltop&blast_rank=1&RID=DH1K9TYJ01R) \| \| --- \| |
| FaPIN | GCCATACTATCAGACGCA | AACAGACCATCACACCAG | [XM_004299482](http://www.ncbi.nlm.nih.gov/nucleotide/470127132?report=genbank&log$=nucltop&blast_rank=1&RID=DGA95ZM6015) |
| FaYUCCA | CACTCGTTGTCAGAGATACAGTGTG | TAATCTCTTGATTGCCGGACGTAC | JF898837 |
| SlSUT | TAC AGT TTC GCA TCA CCG AC | AAC TCC CGG AGA AAG AAG AG | NM_001302901 |
| SlSS | GGCAGAGATGAAGAAGATGT | CGACAACAGTCAGACCAA | NM_001247726 |
| SlSPS | CGGTGGATGGCAAAACG | GGCAATCGGCCTCTGGT | AB051216.1 |
| SlAI | AGGTGTTTCTCAGGGAGTC | GTAAGCCGTTCTTTGCCA | NM_001247914 |
| FaSUT | CTACAGCGACCGTAACACC | ACAACAAATACAGCCACAGC | XM_004291847 |
| FaSS | TTATCCCTCGCATTCTTATT | CAATTCCCTTCTCGGTTCTA | AB275666.1 |
| FaSPS | GAATGTCCCTATGTTATTTACTGG | TCCTGTCTGGTGCTGGTTAT | AB267869.1 |
| FaAI | CCGATACCGAGTCCGATGA | TTCAACAACTGCTCCTGCTT | AB275667.1 |
| FaEG1 | AACTTCGCTATCGTTCCTGCTT | CCACCTAATGTAAAACCCACTGT | AJ006348.1 |
| FaXTH1 | TGGCAATGAGATTCAGCTCCACC | TCTGATCCCACCACCTCTTACCCT | GQ367550.1 |
| SlSAND | TTGCTTGGAGGAACAGACG | GCAAACAGAACCCCTGAATC | SGN-U316474 |
| FaActin | TGGGTTTGCTGGAGATGAT | CAGTAGGAGAACTGGGTGC | AB116565 |

**Figure S1**


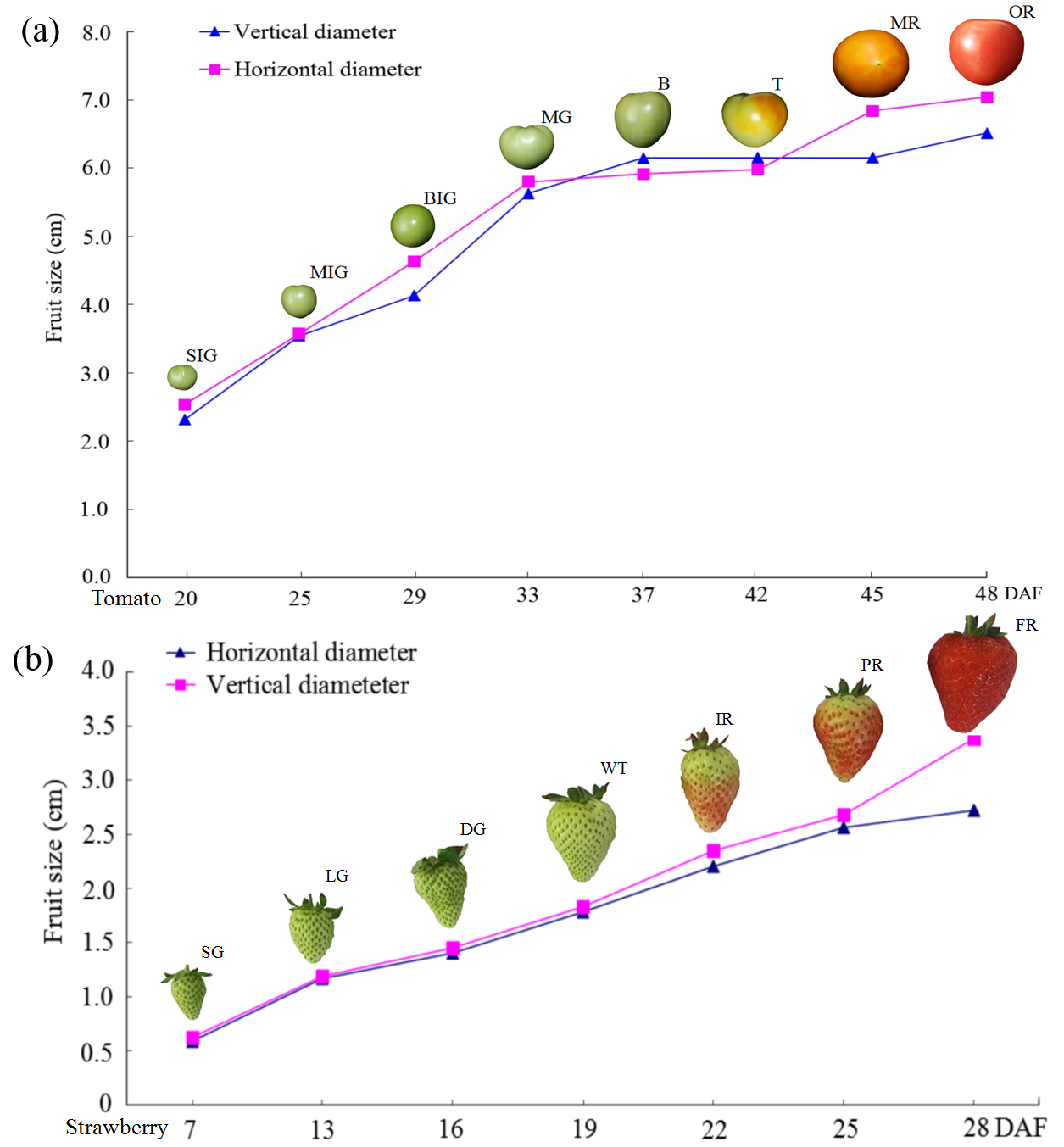


Figure S1 Morphological and physiological changes in the receptacle of strawberry fruit and tomato fruit during developmental processes. (A) Fruit developmental processes were divided into the following seven stages for strawberry: (SG (Small green), LG (Large green), DG (Degreening), WT (White), IR (Initial red), PR (Partial red), and FR (Full red)) for 7, 13, 16, 19, 22, 25, and 28 days after anthesis. (B) Eight stages for tomato: Small immature green (SIG), Middle immature green (MIG), Big immature green (BIG), Mature green (MG), Breaking (B), Turning (T), Mature red (MR) and over red (OR) at about 20, 25, 29, 33, 37, 42, 45 and 48 days after anthesis, respectively.

**Figure S2**


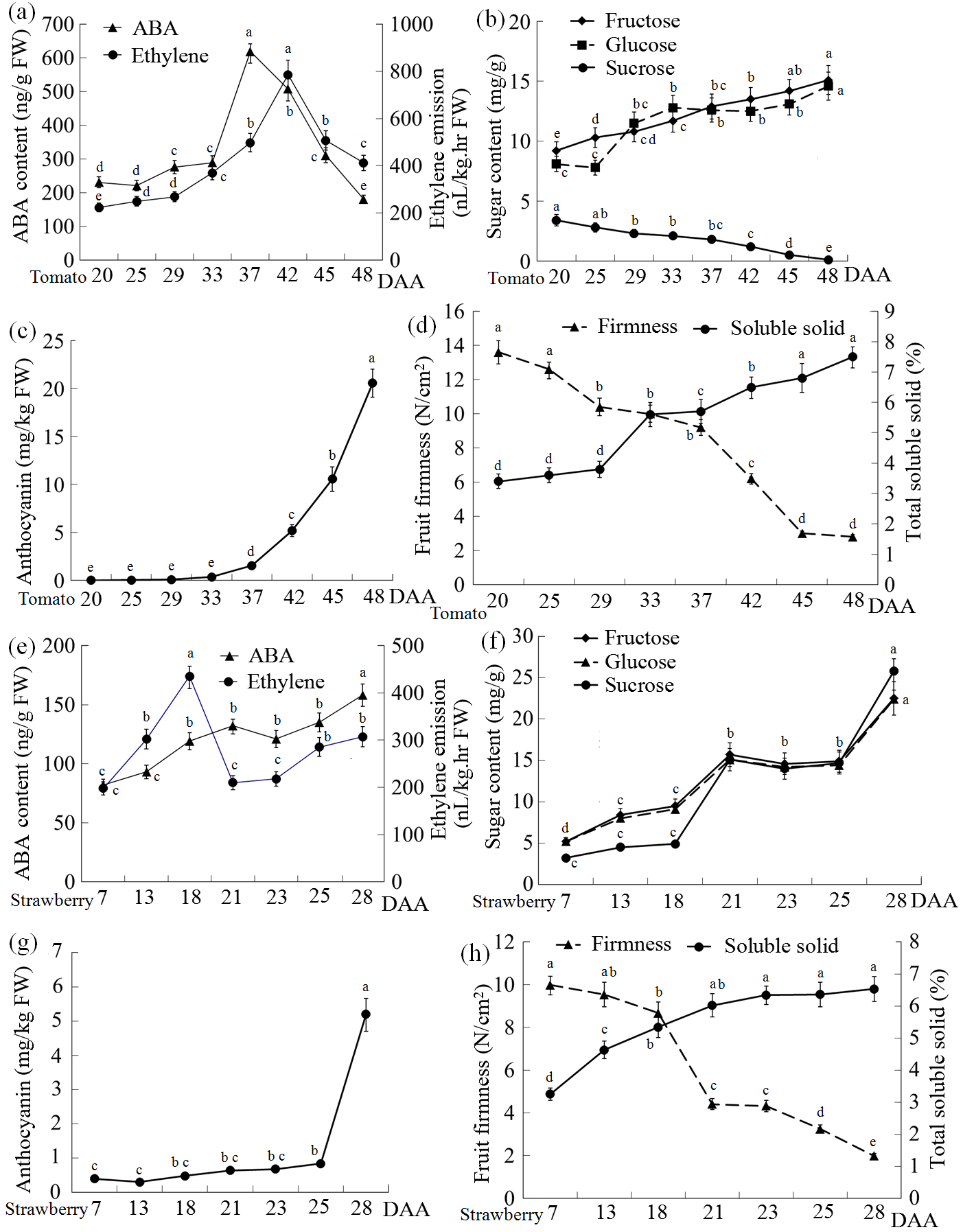


Figure S2 Physiological change in tomato and strawberry fruit. Changes of (A) abscisic acid (ABA) content and ethylene content, (B) three soluble sugars content, and (C) anthocyanin content, and (D) fruit firmness during tomato fruit development. Changes of (E) abscisic acid (ABA) content and ethylene content, (F) three soluble sugars content, (G) anthocyanin content, and (H) fruit firmness during strawberry fruit development. Values were means +SD of four biological replicates. Different letters indicated a statistical difference at *P*<0.05 as determined by Duncan’s multiple range test.

**Figure S3**


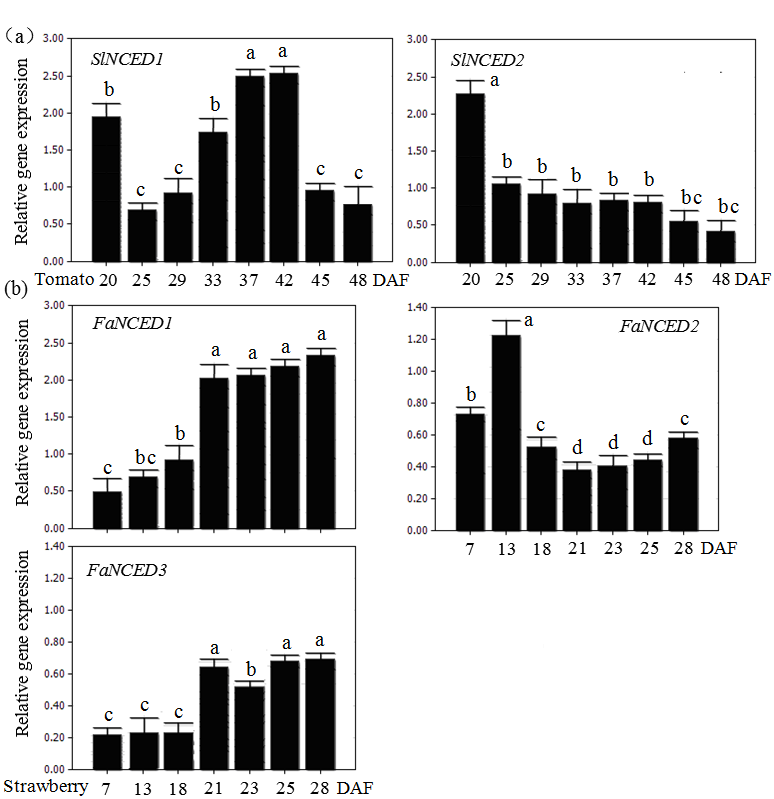


Figure S3 qRT-PCR of *NCEDs* genes expression level during tomato and strawberry fruit development. (A) The two *NCEDs* genes: *SlNCED1* (Genbank access number: Z97215), *SlNCED2* (EU912387) genes expression level during tomato fruit development, and *SlSAND* gene was used as an internal control. (B) Three *NCEDs* genes: *FaNCED1*(JX013944), *FaNCED2* (JX013945), and *FaNCED3* (JX013946) genes expression level during strawberry fruit development, and *FaActin* gene was used as an internal control. Error bars represent SE (n = 3). Different letters indicated a statistical difference at *P*<0.05 as determined by Duncan’s multiple range test.

**Figure S4**


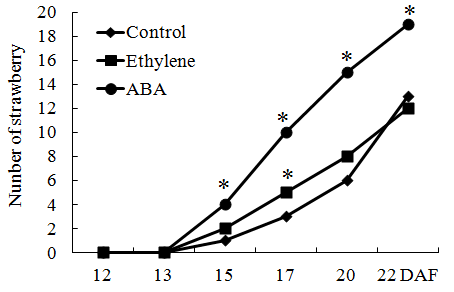


Figure S4 The effects of exogenous ABA (50 µm) and ethephon (50 µm) on strawberry fruit ripening process. Fruits were treated with these exogenous compounds at 12 d after flowering. Colored fruits were calculated for each observation time by counting the numbers of fruits showing any degree of color development. It was repeated three times and got the similar result. Asterisks indicated statistically significant differences between treatment and control at P < 0.05 as determined by Student’s test.

**Figure S5**


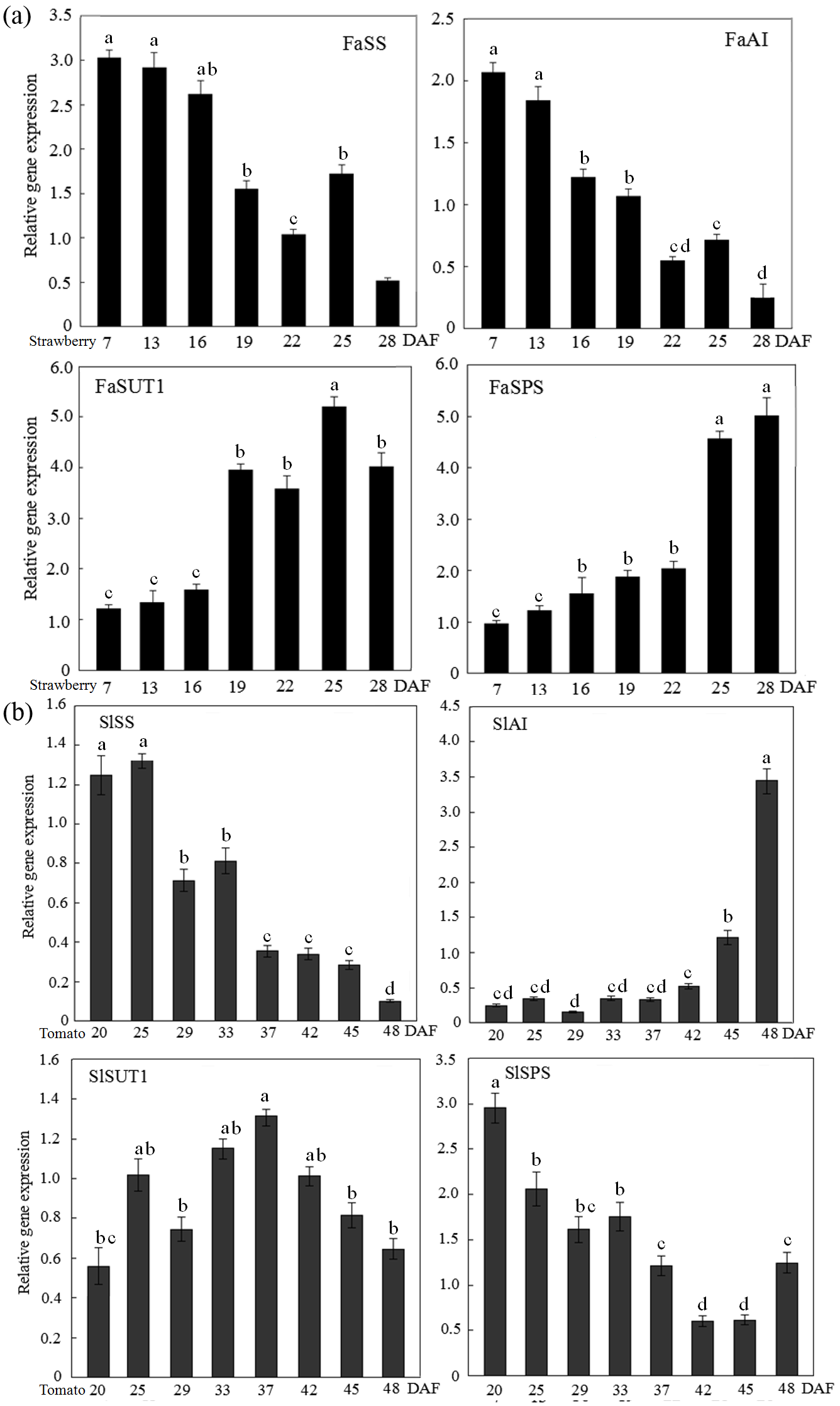


Figure S5 qRT-PCR of sucrose metabolism-related genes expression level in the process of tomato and strawberry fruit development. Total RNA was isolated from the fruits of various developmental stages of (A) tomato and (B) strawberry, and the genes expression level of *SS*, *AI*, *SUT1*, and *SPS* was determined. *SlSAND* and *FaActin* genes were used as a internal control for tomato and strawberry genes expression determination, respectively. *SS*: Sucrose synthase gene; *AI*: Acid invertase gene; *SUT1*: Sucrose transporter gene; *SPS*: Sucrose phosphate synthase gene. Error bars represent SE (n = 3). Different letters indicated a statistical difference at *P*<0.05 as determined by Duncan’s multiple range test.

**Figure S6**


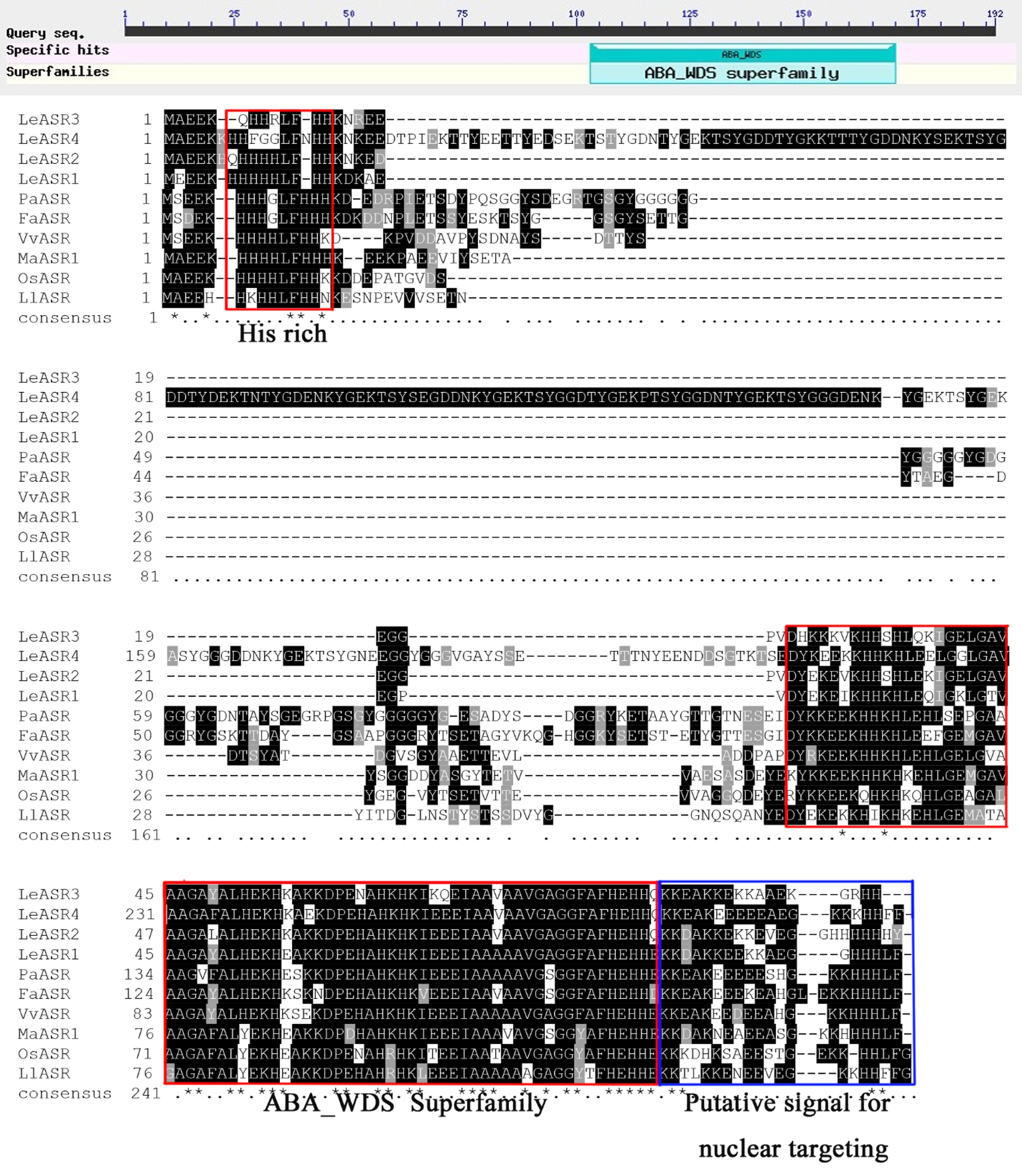


Figure S6 Amino acid sequence alignment of tomato SlASR and strawberry FaASR with other plant ASR proteins. Tomato SlASR and strawberry FaASR were aligned with Apricot (*Prunus armeniaca*, U93164, PaASR), Grape (*Vitis vinifera*, AF281656, VvASR), Plantain (*Musa acuminate*, AY628102, MaASR1), Rice (*Oryza sativa*, KF916487, OsASR), Lily (Lilium longiflorum, AY101194, LlASR). Conserved residues were shaded in black. Multiple alignments were done by CLUSTALW and viewed with BOXSHADE program, and then manually edited. One Zn-binding His-rich region and two Ala-rich regions at N terminus were underlined. The ABA/WDS motif was indicated with blue rectangle. A putative nuclear targeting signal (KKEDKEEAEEASGKKHHH) at C terminus was marked with red rectangle.

**Figure S7**


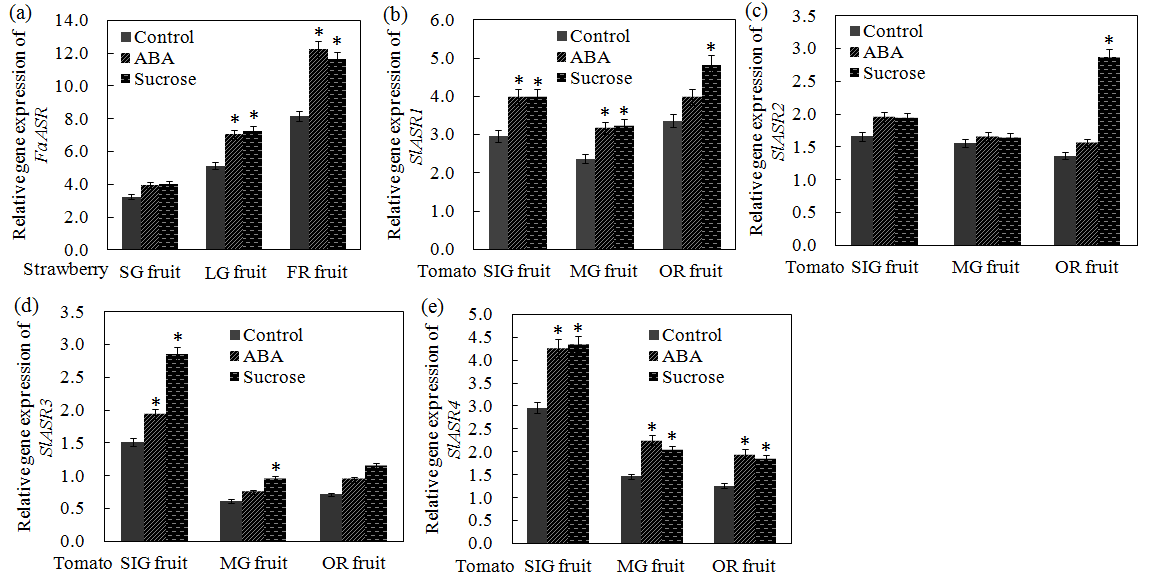


Figure S7 Determination of *ASR* gene expression level induced by ABA and sucrose in strawberry and tomato. qRT-PCR was performed to determine of (A) *FaASR* gene expression level in strawberry, and four members of *SlASRs*: (B) *SlASR1*, (C) *SlASR2*, (D) *SlASR3*, and (E) *SlASR4* gene expression level in tomato. Strawberry of SG: Small green fruit; LG: Large green fruit; FR: Full red fruit. Tomato of SIG: Small immature green fruit; MG: Mature green fruit; OR: Over red fruit. *FaActin* and *SlSAND* genes were used as the internal control for strawberry and tomato, respectively. Vertical bars represented standard deviations (SD) of means (n = 3). Asterisks indicated statistically significant differences at P < 0.05 as determined by Student’s test. Control: Deonized water treatedfruit; ABA: 50 µM ABA treated fruit; Sucrose: 100 mM sucrose treated fruit.

**Figure S8**


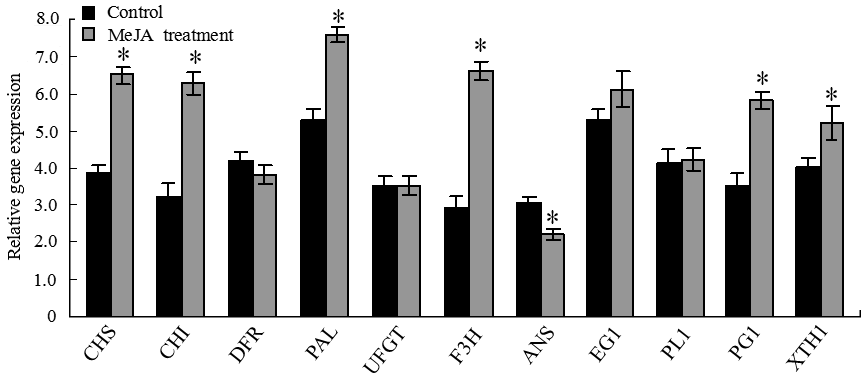


Figure S8 The influence of jasmonic acid on the fruit ripening-related genes. The methyl jasmonic acid was used to inject the LG (33 d) stage of strawberry fruit, and then total RNA was extracted to determine the gene expression level, and deionized water was used as control. Eleven genes were analyzed: *Exp1*/2: expansion1/2; *Cell*: Cellulase; *PG*: polygalacturonase; *PL:* pectate lyase; *CHS*: chalcone synthase. *FaActin* mRNA was used as the internal control. Error bars represented SE (n = 3). Asterisks indicated statistically significant differences at P < 0.05 as determined by Student’s test.

**Figure S9**


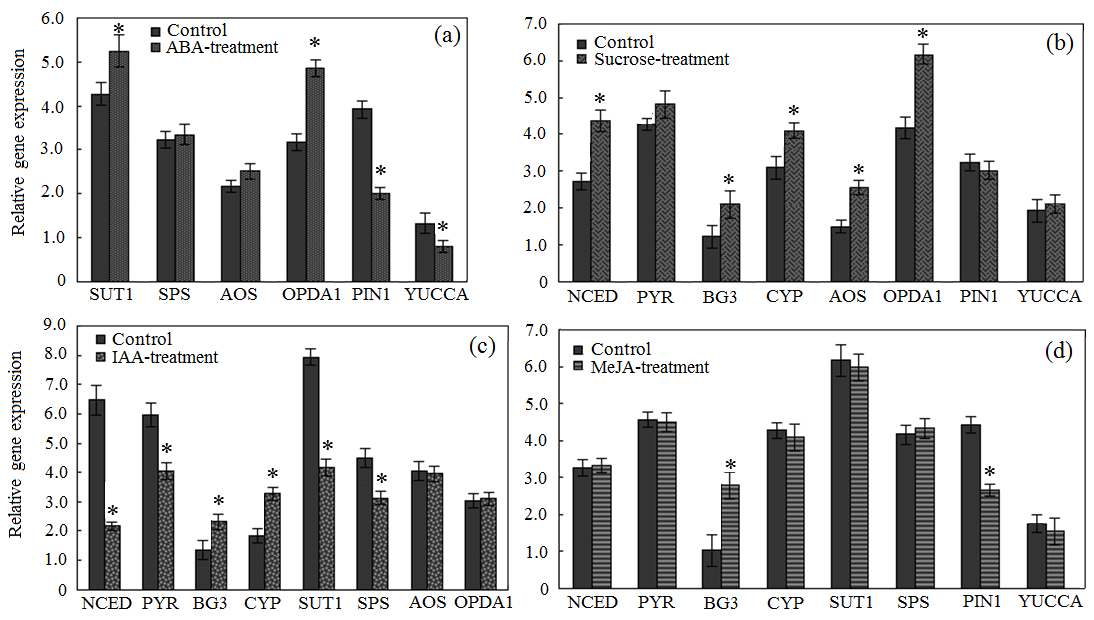


Figure S9 Determination of the relationship of four ripening-related factors: ABA, JA, IAA, and sucrose in strawberry. Total RNA was extracted from (A) ABA, (B) sucrose, (C) IAA, and (D) JA treated strawberry fruits, their associated genes expression level was measured by qRT-PCR. Genes expression level was normalized with the internal *FaAction* gene. The error bars represent the standard error (n=3). Asterisks indicated statistically significant differences at P < 0.05 as determined by Student’s test. *NCED*: 9-cis-epoxycarotenoid dioxygenase; *BG*: β–glucosidase gene; *CYP707A*: ABA 8′-hydroxylases gene; *PYR*: Pyrabatin Riesistance; *SUT1*: Sucrose transporter gene; *SPS*: sucrose phosphate synthesis gene; *AOS*: Allene oxide synthase; *OPDA1*: 12-oxo-phytodienoic acid; *PIN*: auxin transporter gene; *YUCCA*: flavin monooxygenase gene.

**Figure S10**


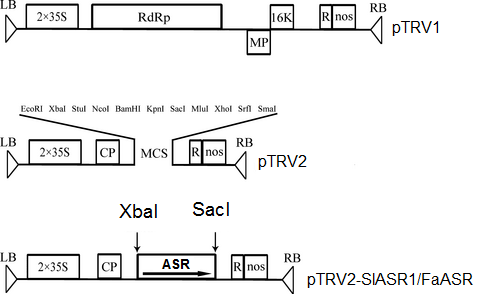


Figure S10 Construction of pTRV1, pTRV2 and pTRV2-derivative pTRV2-*SlASR1* or pTRV2-*FaASR*. The TRV-based virus-induced gene silencing vectors were as described by Fu *et al* (2005). TRV cDNA clones were placed between duplicated CaMV 35S promoters and the nopaline synthase terminator in a T–DNA vector. pTRV2-*ASR* (sense orientation) was constructed to assess the ability of TRV vectors to suppress expression of the *SlASR1* or *FaASR* gene in tomato or strawberry fruits. RdRp, RNA-dependent RNA polymerase; 16K, 16 kDa cysteine-rich protein; MP, movement protein; CP, coat protein; LB and RB, left and right borders of T–DNA, respectively; R, self-cleaving ribozyme; MCS, multiple cloning sites.

**Figure S11**


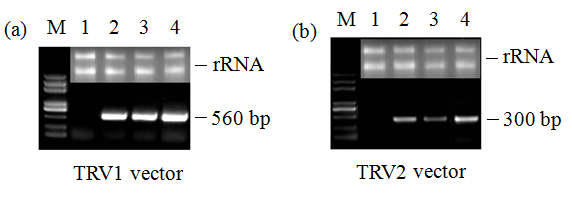


Figure S11 RT-PCR analysis of TRV expression in fruits. Two weeks after infiltration, virus vector gene expression of both (B) 560 bp pTRV1 and (C) 300 bp pTRV2 was detected in fruits infiltrated with *Agrobacterium* containing TRV (lane 2: fully red fruits; lane 3, 4: chimeric fruits), but not detected in fruits infiltrated with *Agrobacterium* alone (lane 1).

**Figure S12**


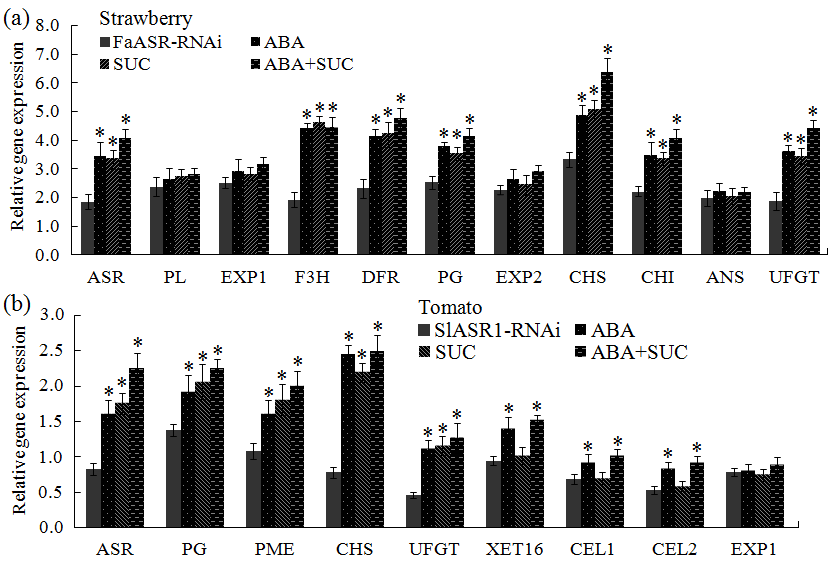


Figure S12 Effect of ABA, sucrose, and ABA+sucrose on the cell wall and anthocyanin metabolism gene expression levels in the *ASR*-RNAi fruit of strawberry and tomato. Ten fruits were used for each treatment, ABA (50 μM), sucrose (SUC, 100 mM), or ABA(50 μM)+sucrose(SUC,100 mM) was injected into the un-color part of ASR-RNAi fruit, respectively, 8 hours later the total RNA was isolated, and qRT-PCR was performed to determined genes expression level in (A) strawberry and (B) tomato. Vertical bars represented standard deviations (SD) of means (n = 3). Asterisks indicated statistically significant differences between treatment and control at P < 0.05 as determined by Student’s test.

*CHS*: chalcone synthase; *CHI*: chalcone isomerase; *F3H*: flavanone-3 –hydroxylase; *DFR*: dihydroflavonol 4-reductase; *ANS*: anthocyanidin synthase; *PG*: polygalacturonase; *PL*: pectate lyase; *EXP*: expansin protein; *UFGT*: Uridine diphosphate glucose-flavonoidglucosyltransferase; *CEL*: Cellulase; *XET16*: xyloglucan endo-transglycosylase; *PME*: pectin methylesterase.

**Figure S13**


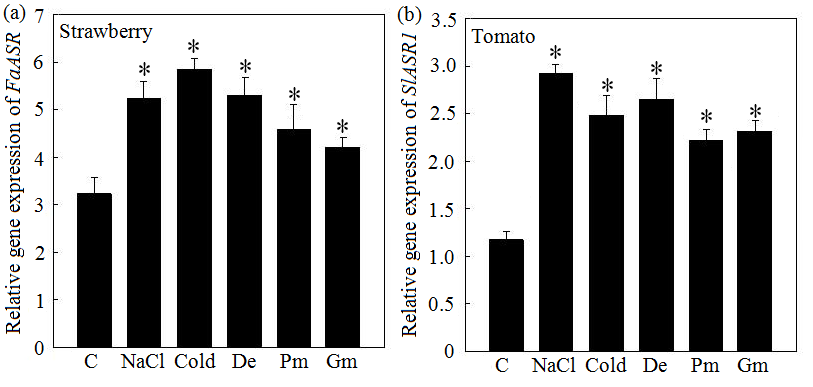


Figure S13 The abiotic and biotic stress on the *ASR* gene expression level of strawberry and tomato fruit. The strawberry and tomato fruits that were 28 days and 48 days after flowering were used, respectively. The method of treatment was performed as described in the material. The qRT-PCR method was presented in the main text, and normalization of the *ASR* expression was according to the reference genes of *SlSAND* gene (Accession number: SGN-U316474) for tomato and *FaActin* gene (AB116565) for strawberry. Vertical bars represented standard deviations (SD) of means (n = 3). Asterisks indicated statistically significant differences at P < 0.05 as determined by Student’s test.

C: Untreated red fruit; De: dehydration red fruit; Pm: powdery mildew infected red fruit Gm: gray mold infected red fruit.
